# *H19* Antisense RNA Can Up-Regulate *Igf2* Transcription by Activation of a Novel Promoter in Mouse Myoblasts

**DOI:** 10.1371/journal.pone.0037923

**Published:** 2012-05-25

**Authors:** Van Giang Tran, Franck Court, Anne Duputié, Etienne Antoine, Nathalie Aptel, Laura Milligan, Françoise Carbonell, Marie-Noëlle Lelay-Taha, Jacques Piette, Michaël Weber, Didier Montarras, Christian Pinset, Luisa Dandolo, Thierry Forné, Guy Cathala

**Affiliations:** 1 Institut de Génétique Moléculaire de Montpellier, UMR 5535 CNRS-Université Montpellier II, Montpellier, France; 2 Molecular Genetics of Development Unit, Department of Development Biology, URA CNRS 2578, Institut Pasteur, Paris, France; 3 ISTEM/CECS, Evry, France; 4 Genetics and Development Department, INSERM U567, CNRS UMR 8104, University Paris Descartes, Institut Cochin, Paris, France; UMDNJ-New Jersey Medical School, United States of America

## Abstract

It was recently shown that a long non-coding RNA (lncRNA), that we named the *91H* RNA (i.e. antisense *H19* transcript), is overexpressed in human breast tumours and contributes in *trans* to the expression of the *Insulin-like Growth Factor 2* (*IGF2*) gene on the paternal chromosome. Our preliminary experiments suggested that an *H19* antisense transcript having a similar function may also be conserved in the mouse. In the present work, we further characterise the mouse *91H* RNA and, using a genetic complementation approach in *H19* KO myoblast cells, we show that ectopic expression of the mouse *91H* RNA can up-regulate *Igf2* expression in *trans* despite almost complete unmethylation of the Imprinting-Control Region (ICR). We then demonstrate that this activation occurs at the transcriptional level by activation of a previously unknown *Igf2* promoter which displays, in mouse tissues, a preferential mesodermic expression (Pm promoter). Finally, our experiments indicate that a large excess of the *H19* transcript can counteract *91H*-mediated *Igf2* activation. Our work contributes, in conjunction with other recent findings, to open new horizons to our understanding of *Igf2* gene regulation and functions of the *91H*/*H19* RNAs in normal and pathological conditions.

## Introduction

Long non-coding RNAs (lncRNAs) are major components of the mammalian transcriptome (for a review, see ref. [1]). Recent efforts to better characterize such transcripts revealed that they play important roles in both oncogenic and tumour suppressive pathways [2]. LncRNAs display a myriad of molecular functions [3], from chromatin remodelling (*ANRIL*, *HOTAIR*, *Xist*) [4], [5], [6], [7] and modulation of alternative splicing (*Zeb2/Sip1* gene locus) [8], to RNA metabolism (*1/2-sbs* and *HULC* RNAs) [9], [10] and generation of micro- and small-RNAs (*MEG3*/*Gtl2* and *MALAT1* transcripts) [11], [12]. They also have a great variety of forms: most of them are generated by the RNA polymerase II, but some are synthesized by the RNA Pol III (BC200 RNA) [13]; moreover, while most are poly-adenylated, many lncRNAs remain unpoly-adenylated [14] like, for example, the natural Sense-Antisense transcripts (SAT) [15] which are known to overlap each other and are co-ordinately expressed [16].

Several lncRNA are also produced from imprinted genes, whose expression is depending on the parental origin of the chromosome. Among them, the *Airn* and *Kcnq1ot1* transcripts have been shown to “coat” the imprinted locus on the paternal chromosome from which they are expressed. Interestingly, both transcripts are known to interact with the histone H3 Lysine 9 methyltransferase G9a and to repress multiple genes in *cis* on the paternal chromosome [17]. Finally, two genes encoding imprinted lncRNAs map downstream the *Insulin-like Growth Factor 2* gene (*Igf2*) : one is the recently described *PIHit* [18] and another is the *H19* gene.

Since its discovery, twenty years ago [19], the function of the *H19* gene remains enigmatic. *H19* gene silencing is associated with the appearance of Wilms’ tumours in the Beckwith-Wiedemann syndrome [20], [21]. Furthermore, ectopic expression of the *H19* gene in human embryonic tumour cell lines leads to loss of clonogenicity and reduced tumourigenicity in *nude* mice [22]. It was recently confirmed that, in the mouse, *H19* acts as a tumour suppressor [23]. However, several studies have also shown that the *H19* RNA can accumulate in cancer cells and tumours [24], [25], [26], [27], [28] and it has been considered as an oncofetal RNA by some authors [29]. The gene encodes an untranslated RNA which is expressed only when maternally inherited. Monoallelic expression of *H19*, like that of the neighbouring oppositely imprinted *Insulin-like growth factor-2* (*Igf2*) gene, depends on the paternal-specific DNA methylation of an Imprinting-Control Region (ICR) located between 2 and 4 kb upstream of the *H19* gene [30]. This methylation is acquired during male gametogenesis and prevents the binding of CTCF, an insulator protein. On the unmethylated maternal allele, CTCF is bound to the ICR and creates a boundary which prevents interactions between enhancers, located downstream of the *H19* gene, and the *Igf2* gene [31].

While the mechanisms of imprinting at the *Igf2/H19* locus have focused much attention, very little is known about transcriptional regulation of the expressed *Igf2* and *H19* alleles. The two genes are tightly co-regulated during mouse embryonic development and are repressed shortly after birth in most tissues. Both genes belong to a network of coexpressed imprinted genes (Imprinted Gene Network, IGN) that may control embryonic growth in the mouse [32]. Recently, the non-coding *H19* RNA was shown to contribute to the *trans* regulation of at least 9 genes of the IGN [33]. However, whether the *H19* transcript acts through direct or indirect mechanisms and which step of gene expression is affected by such a regulation have not yet been investigated. Interestingly, we recently discovered in human that an antisense *H19* transcript, named the *91H* RNA (or *H19os* for “*H19* opposite strand” transcript), augments in *trans* the paternal *IGF2* expression which is known to favour tumour progression. In agreement with this notion, the *91H* RNA is a large nuclear transcript which accumulates in breast tumours by RNA stabilization [34]. Preliminary experiments indicated that the antisense *H19* transcript is evolutionarily conserved and expressed during the perinatal period in the mouse. In this work, we present further insights about the function of the mouse *91H* and *H19* transcripts. Using a genetic inactivation/complementation approach in cultured murine myoblasts, we show that ectopic expression of *91H* RNA is sufficient to *trans*-activate *Igf2* at the transcriptional level, despite hypomethylation of the *H19* ICR. Interestingly, this *trans*-activation occurs via a novel *Igf2* promoter (*Igf2* Pm). Our experiments also indicate that a large excess of *H19* RNA seems to counteract this effect. Globally, our work suggests that *H19* sense and antisense RNAs are antagonist *trans* riboregulators of *Igf2* transcription.

## Results

### Characterization of the Mouse *91H* RNA

**Figure 1:**
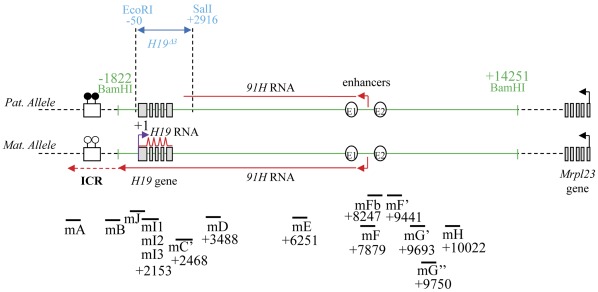
Map of the mouse *H19/91H* region showing the PCR amplicons used in RT-qPCR experiments. The region corresponding to the sequence removed by the H19^Δ3^ deletion in the *H19* KO myoblasts (see below) is indicated in blue. The *H19/91H* insert transfected into the *H19* KO myoblast cells is also shown in the figure (green lane). The insert is a 16 kb BamHI-BamHI fragment encompassing the *H19* endodermic enhancers and the whole *H19* gene. PCR amplicons used to quantify the ectopic RNAs are indicated (*H19* RNA, mI1-mI3, mJ and mC’). DNA methylation is indicated by black lollipops and RNAs are depicted in red. Positions of restriction sites and PCR amplicons used for real-time PCRs are indicated relative to the *H19* transcription start site. The mA and mB PCR amplicons have been used in a previous study [34] and are indicated here solely for clarity of our PCR nomenclature. For primer sequences see Table S1.

Preliminary experiments showed that an *H19* antisense transcript, called the *91H* RNA, that controls *IGF2* gene expression in human breast cancer cells is conserved in the mouse [34]. The *91H* RNA is a short-lived nuclear transcript which is co-regulated with the *Igf2* and *H19* genes, during the perinatal period. Our first aim was to further characterize the mouse transcript before investigating the mechanisms by which it may exert its function. We previously determined in mouse liver the transcriptional orientation of the *91H* RNA upstream of the *H19* endodermic enhancers (mE region, see Figure 1 and Figure S2B of ref. [34]). Since the human *H19* antisense RNA is initiated further downstream, in the intron 1 of the *MRPL23* gene [34], we investigated in the mouse the intergenic region located downstream of the *H19* endodermic enhancers. In the heart, strand-specific RT-qPCR quantifications showed that the antisense transcript is mostly initiated downstream of the *H19* enhancers (data not shown), reminiscent to the human situation [34]. However, in the mouse liver, we found only little antisense relative to the sense transcript downstream of the enhancers (data not shown). Since, upstream the enhancers, we found substantial amounts of antisense transcript [34], we can conclude that, opposite to the situation in the heart, in liver, most of the *91H* RNA is initiated within the endodermic enhancer region. As shown previously [34], [35], within the *H19* ICR and its upstream sequences short non-coding transcripts are initiated in both sense and antisense directions on both parental alleles, thus impairing further characterisation of the 3′ end of the *91H* RNA.

**Figure 2:**
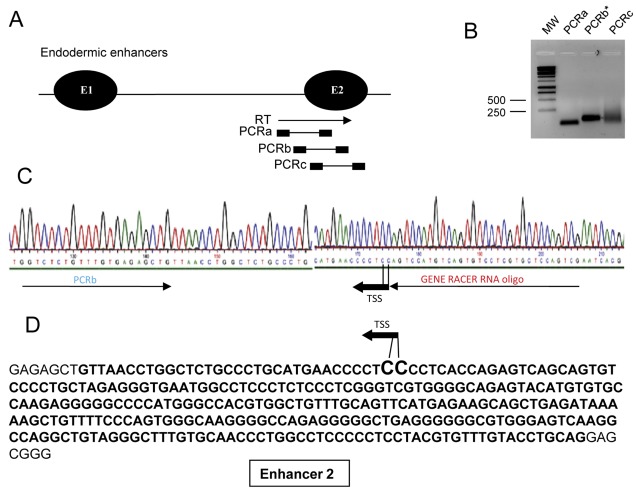
Characterisation of TSS of the endogenous mouse *91H* RNA. 5′RACE experiment was performed on unpolyadenylated and capped RNA from 7 days-old mouse liver. (A) The RT Primer was designed in the mFb region (Figure 1) and a band was successfully amplified by nested PCRs. The RT primer corresponds with the forward primer of PCRa and nested PCR reactions were performed using the GeneRacer DNA oligonucleotide as reverse primer. (B) Ethidium bromide staining of an agarose gel showing PCRs product obtained from amplifications indicated above (MW: Molecular Weight). Sequencing of PCRa and PCRc products showed that these bands correspond essentially to unspecific amplifications while PCRb correspond to the TSS of the *91H* RNA. (C) Electrophoregram of the sequenced 5′RACE product amplified from the capped RNA fraction (PCRb). This sequence identified a unique Cap site located in the endodermic enhancer 2 at position chr7:149,755,206 or chr7:149,755,207 on mouse July 2007/ mm9 Assembly. Due to the presence of a C residue at the end of the GeneRacer RNA oligonucleotide primer and/or the possibility that the last C residue may derive from the cap of the RNA, the exact position of the TSS remains ambiguous between two consecutive C residues found in the mouse genome sequence. (D) The sequence of the endodermic enhancer 2 is indicated in bold. The position of the TSS of the *91H* RNA is indicated (black arrow).

5′RACE experiment from the capped fraction of non-polyadenylated d7 mouse liver RNAs then mapped the Transcription Start Site (TSS) of the *91H* RNA more precisely within the endodermic enhancer 2 (position chr7: 149,755,206 or chr7: 149,755,207 on mouse July 2007/ mm9 Assembly) (Figure 2).

Finally, using mice carrying a maternal deletion of the *H19* transcription unit (*H19* ^Δ*3*^ mutant) [36] and a primer pair specific of the *wild-type* allele (mJ PCR amplicon, that amplifies through the 5′ end of the *H19* ^Δ*3*^ deletion over the *H19* transcription start site, Figure 1), we assessed by RT-qPCR that no paternal allele-specific transcript can be detected immediately upstream of the *H19* gene (data not shown) while it can be detected further downstream on this allele [34]. Therefore, as previously suggested [34], the mouse *91H* RNA transcription stops within the *H19* gene region on the paternal allele (Figure 1).

### *H19* KO Myoblasts Display Low ICR Methylation and Weak *Igf2* Expression Levels

**Figure 3:**
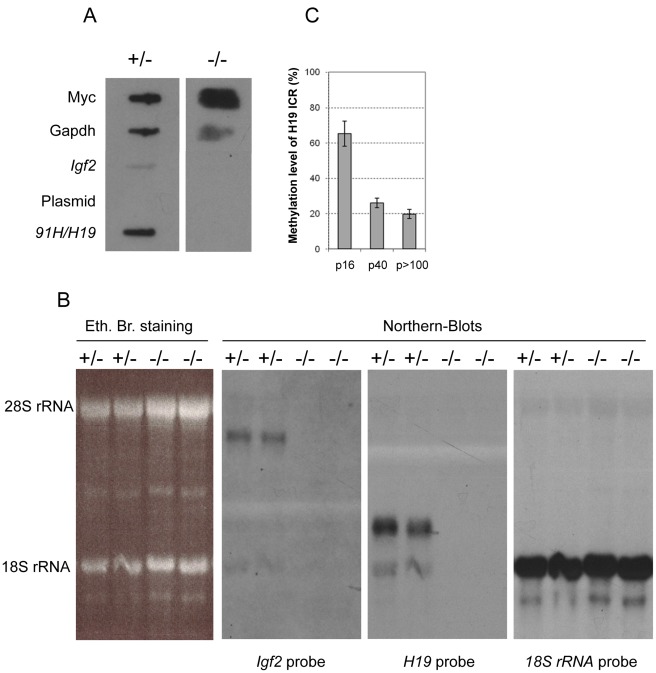
Transcriptional and steady state *Igf2* RNA levels in control and *H19* KO myoblasts. *H19* KO myoblats (−/−) have been compared to cells with a paternally inherited deletion of the *H19* transcription unit (control paternal heterozygous myoblats, +/−). These two cell lines have identical genetic background and were both harvested after identical late passage numbers (passage 40). (A) Run-on analysis of *Igf2* and of the *91H/H19* transcripts. *Igf2* transcriptional activity is detected only in control myoblasts (+/−). (B) Northern-blot analyses of *Igf2* mRNA and *H19* transcript levels showing two lanes corresponding to two separate samples for each cell type. The same membrane was sequentially hybridized with *Igf2*, *H19* and 18S rRNA probes. The left panel shows an ethydium bromide staining of the agarose-formaldehyde gel before transfer. Note that the *Igf2* transcripts are detected only in control cells (+/−). (C) Methylation levels were determined at a BceAI methylation sensitive restriction site located within the CTCF recognition site 2 of the *H19* ICR in H19 KO myoblasts at the indicated passage numbers.

We previously showed that the mouse *H19* RNA is a negative *trans*-regulator of *Igf2* mRNA levels [33] while, in the human, the *91H* RNA augments paternal *IGF2* expression levels [34]. Furthermore, a study by Wilkin *et al.* suggested that the activity linked to the *91H* RNA may be restricted to the antisense strand of the *H19* transcription unit [37]. To elucidate the function of the sense/antisense *H19* transcripts, we generated a mouse myoblast cell line from homozygous *H19* ^Δ3^ KO mice [36] (*H19* KO myoblasts −/−). These cells express an endogenous *91H* RNA which is truncated within the *H19* transcription unit. Therefore, we suspected that this truncated *91H* RNA would not be functional and that *Igf2* expression may be affected in this cell line. Remarkably, we showed that, as for the classical C2C12 myoblast line, *H19* KO myoblasts can differentiate into myotubes upon 3 days of serum starvation (Figure S1). Using the mE and mF PCR amplicons (Figure 1), we then showed that the endogenous truncated *91H* RNA displays a similar level of expression compared to the native *91H* RNA in C2C12 myoblasts (Figure S2A). As found for tissues [34], the endogenous truncated *91H* RNA levels are very low as compared to that of the sense *H19* transcript (about 10^3^/10^4^ lower than *H19* RNA levels observed in C2C12 myoblasts). Interestingly, in early passages of cell culture, *H19* KO myoblasts displayed substantial *Igf2* expression levels, as observed in the muscle of *H19* ^Δ*3*^ animals [36] (data not shown). However, in later passages, we found that *Igf2* expression levels were very weak in undifferentiated cells and could only be detected by RT-qPCR (Figure S2B) but neither inrun-on (Fig. 3A) nor in Northern-Blot (Fig. 3B) experiments.

Run-on and Northern-blot experiments were also performed on myoblasts derived from mice having a paternal inheritance of the *H19* ^Δ*3*^ deletion (control paternal heterozygous myoblasts: +/−). These cells display the same genetic background than *H19KO* myoblasts and they were cultivated under identical experimental conditions (late passages of cell culture). However, they harbour accurate *Igf2* transcription (Figure 3A) and regular steady state *Igf2* mRNA levels (Figure 3B). Finally, *Igf2* dowregulation in *H19KO* myoblasts was correlated with a progressive loss of DNA methylation of the *H19* ICR (CTCF site 2) observed upon increasing passages in cell culture (Figure 3C). Therefore, loss of *Igf2* expression in this cell line appears to be linked to ICR demethylation that may itself result, directly or indirectly, from the deletion of the *H19* transcription unit.

### Isolation of *H19* KO Myoblast Clones that Express Ectopic *91H* and *H19* Transcripts

**Figure 4:**
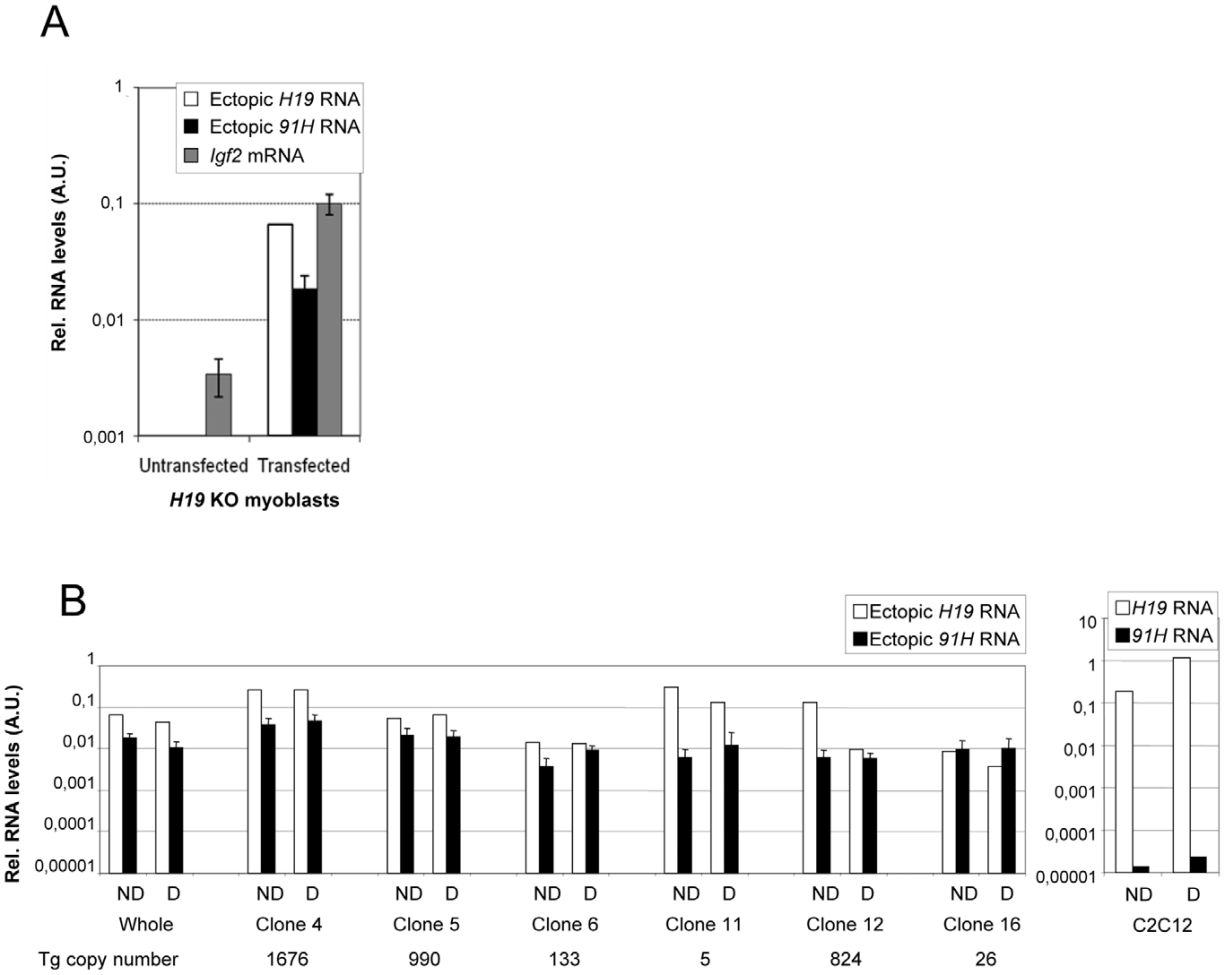
Quantifications of *91H and H19* ectopic RNAs and endogenous *Igf2* RNA levels in transfected *H19* KO myoblast cell lines. (A) The *91H/H19* insert (see Figure 1) have been co-transfected with a hygromicin-resistance plasmid into *H19* KO myoblast cells. Ectopic *91H*, *H19* and endogenous *Igf2* mRNA (Igf2mRNA PCR amplicon) levels were determined by RT-qPCR in undifferentiated *H19* KO myoblasts untransfected or transfected (whole hygromycin-resistant transfected cell population) with the *91H/H19* insert. Results were normalized to *Gapdh* expression levels. (B) Comparison between the *H19* and *91H* ectopic RNA levels. RT-qPCR quantifications were performed in undifferentiated (ND) or differentiated (D). The whole hygromycin-resistant transfected cells (“whole”) and 6 isolated clones (left panel), as well as C2C12 myoblasts (right panel), were analysed. The ectopic *H19* RNA expression levels (open bars) were assessed by using qPCR primers located at *H19* exon-exon junctions (*H19* RNA PCR amplicon). The ectopic *91H* RNA levels (black bars) were quantified with the mC’ and mI1-mI3 PCR amplicons. Error bars correspond to s.e.m. of quantifications obtained with mC’ and the mean of mI PCR amplicons. Detailed data are shown in figure S5 (see also Materials & Methods section and Table S1). Please note that the other PCR amplicons shown in Figure 1 also target the endogenous truncated *91H* RNA produced from the endogenous locus and therefore they could not be used to quantify ectopic transcripts. Sample names and transgene copy numbers are indicated below the histogram.

To investigate the possibility that the effect of the *H19* transcription unit on *Igf2* expression (Figure 3) may depend on antisense sequences complementary to the *H19* gene [34], [37], we then transfected the *H19* KO myoblasts with constructs containing this region with the *H19* gene under the control of a strong promoter (CMV promoter). Unfortunately, despite intensive efforts, such mini-constructs were systematically unable to express significant levels of the transgenes in *H19* KO myoblast cells. However, transfection of a 16 kb BamHI-BamHI restriction fragment encompassing the *H19* endodermic enhancers and the whole *H19* gene (Figure 1), displayed some ectopic expression (Figure 4). This fragment includes the native *H19* and the *91H* endodermic promoters and starts at a BamHI restriction site located 1.8 kb upstream of the *H19* start site, thus excluding the Imprinting Control Region (ICR) of the locus (Figure 1). This fragment was co-transfected with a hygromicin-resistance plasmid into *H19* KO myoblast cells. Interestingly, after hygromicin selection, in addition to ectopic *91H/H19* RNAs, we recovered *Igf2* expression in the whole population of transfected cells (Figure 4A).

We then isolated 16 clones among which 15 displayed expression from the *H19* construct and chose 6 clones for further characterisation (Figure 4B). All transfected clones displayed high ectopic *H19* and *91H* transcript levels (Figure 4B, left panel). For comparison, in wild-type C2C12 myoblast cells, *91H* RNA levels were dramatically lower than *H19* RNA levels (Figure 4B, right panel). Clearly, ectopic *91H* RNA is overexpressed in all transfected cells analysed. However, contrary to the situation in the wild-type C2C12 cells (Figures 4B, right panel), neither the ectopic *91H* nor the ectopic *H19* transcripts are up-regulated during myoblast differentiation (Figure 4B, left panel) suggesting that some regulatory elements are probably missing in the construct used for ectopic expression. 5′RACE experiment performed on total RNA from one of the clone (clone 4) mapped the same TSS found in liver for the endogenous *91H* RNA (see Figure 2) as well as two minor upstream start sites (Figure S3). Finally, using actinomycin D treatments, we definitively validated our experimental system of cellular complementation by showing that, mimicking their endogenous counterparts (Figure S4 and ref. [34], [38]), the ectopic *H19* RNA is very stable, while the ectopic *91H* transcript is much more labile (Figure S4B).

### Ectopic Expressions of *91H* and *H19* RNAs are Oppositely Linked to Induced *Igf2* Transcription

**Figure 5:**
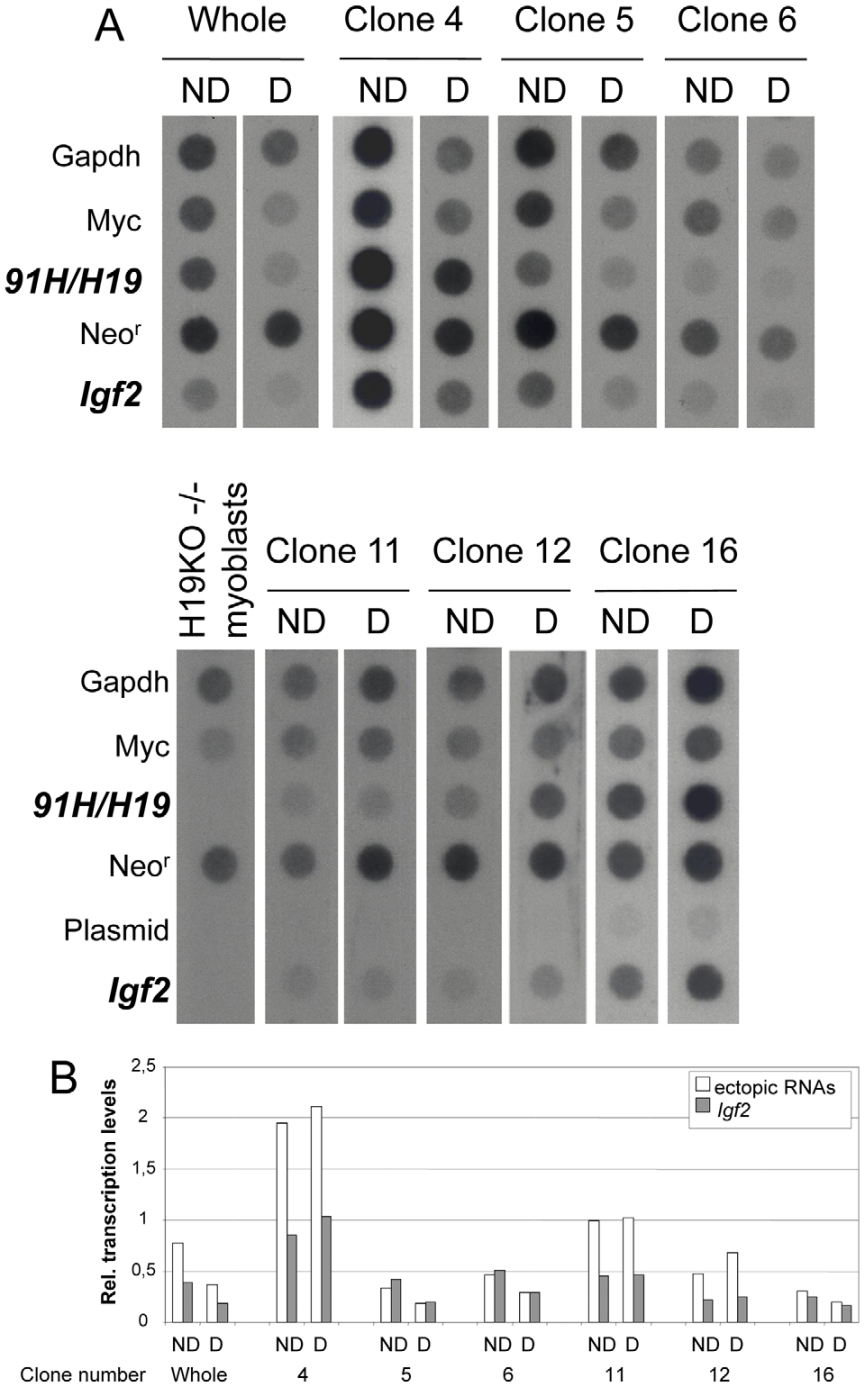
Nuclear Run-on experiments. (A) Autoradiographies of nuclear Run-on experiments on transfected and untransfected *H19* KO myoblasts. Nuclear Run-on experiments were performed as previously described [38] and the α^32^P UTP labelled transcripts were hybridized on filters to denatured plasmids containing the insert DNA of genes indicated on the figure. *91H*/*H19* transcription was assayed using an insert corresponding to the *H19* sequence and *Igf2* with a genomic 2.4 kb BamHI-BamHI DNA fragment encompassing the exon 4-exon 6 region. Such nuclear run-on experiments were performed on undifferentiated (ND) and differentiated (D) cells either on the whole hygromycin-resistant transfected *H19* KO myoblast cell population (“Whole”) and transfected clones. (B) The same filters as those used for the autoradiographies shown in A were used for PhosphorImager quantifications. The ectopic *91H/H19* transcription levels (open bars) were compared to the endogenous *Igf2* transcription levels (black bars). For each hybridized filter, the relative transcription levels were determined for each gene by normalizing to the *Gapdh* transcription level.

To investigate gene expression at the transcriptional level, we performed nuclear run-on experiments on undifferentiated and differentiated transfected *H19* KO myoblasts corresponding to the whole cell population or to the isolated clones (Figure 5A). Using a PhosphorImager, we then quantified the relative *Igf2* transcription levels from the autoradiographies shown in Figure 5A. These experiments showed that *Igf2* transcription is undetectable in untransfected *H19* KO myoblasts, while all transfected clones re-expressed *Igf2* at significant levels (Figure 5B). No correlation was found between *Igf2* transcription levels and the *91H/H19* transgene copy number (R^2^ = 0.0317, data not shown). This demonstrates that *Igf2 trans*-activation occurs at the transcriptional level and suggests that this *trans*-activation depends on ectopic RNA expression.

**Figure 6:**
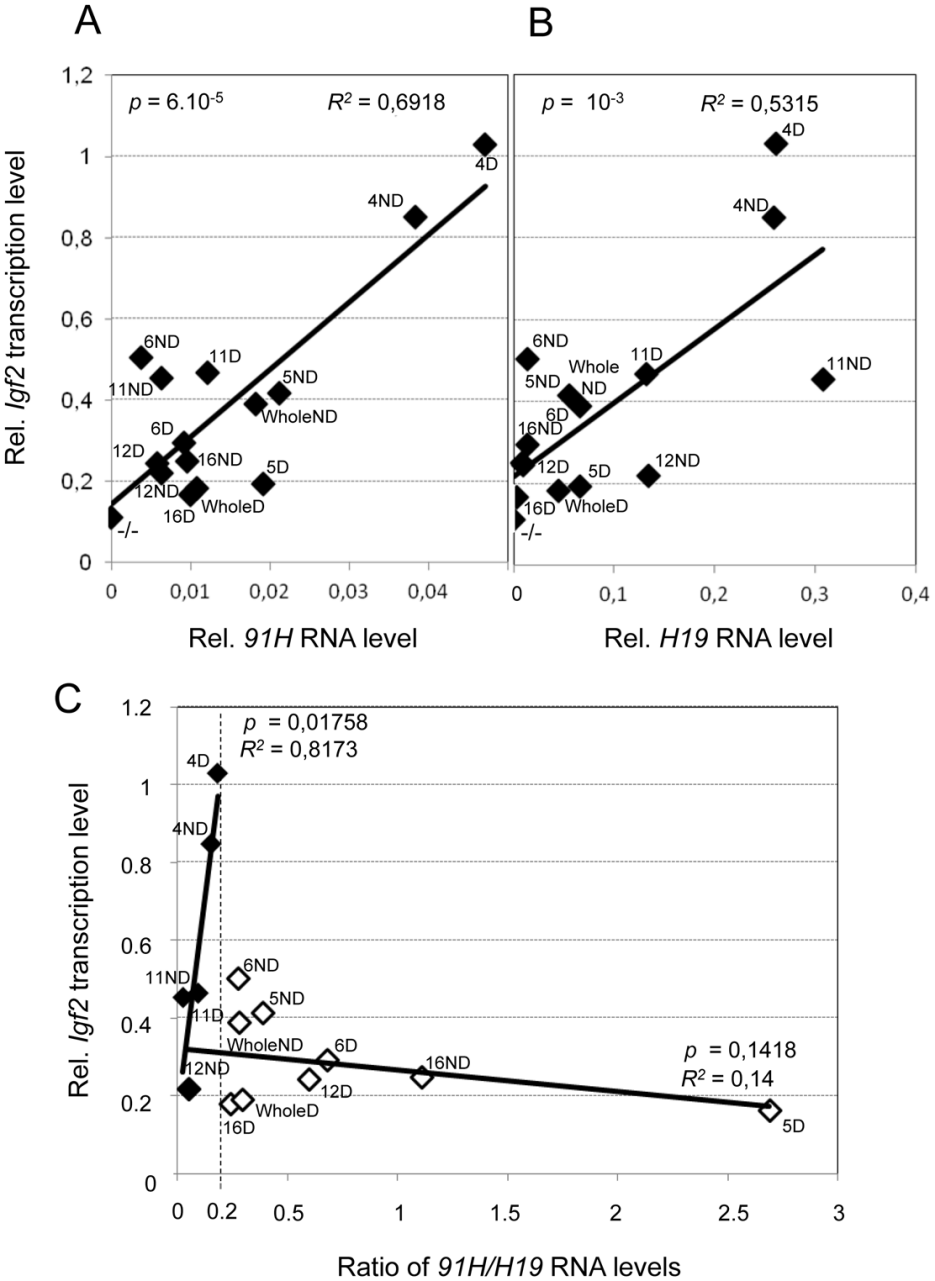
Comparison between the endogenous *Igf2* transcription levels and the steady state levels of the ectopic RNAs. In these graphs, we compared, for each transfected *H19* KO clones, *Igf2* transcription data shown in Figure 5B with the steady state *91H* and *H19* ectopic RNA levels shown in Figure 4B. (A) *Igf2* transcription *versus 91H* RNA levels. (B) *Igf2* transcription *versus H19* RNA levels. In untransfected H19 KO myoblasts (−/−), both *91H* and *H19* are not expressed (RNA levels  = 0) and *Igf2* transcription level is below the “empty plasmid” background (see Fig. 5A) which is inferior to 0.11. (C) *Igf2* transcription level *versus* the ratio of *91H/H19* RNA levels. Clones expressing large amount of ectopic *H19* RNA (clones 4, 11 and 12ND, black diamonds) (Figure 4B) were analysed separately from the others (ratio of *91H*/*H19* RNA levels >0.2; open diamonds). In clones expressing high *H19* RNA levels (black diamonds), the ectopic *H19* RNA level relative to the *91H* RNA level (which leads to a decrease of the *91H/H19* RNA ratio) is inversely proportional to *Igf2* transcription levels (R^2^ = 0.8173).

In agreement with our previous findings in the human [33], [34], *Igf2 trans*-activation is strongly correlated to the ectopic *91H* RNA (*R* ^2^ = 0.6918 ) (Figure 6A). We also observed a positive correlation between *Igf2* transcription and ectopic *H19* RNA (*R* ^2^ = 0.5315) levels (Figure 6B). Both RNAs are produced from the same ectopic copies and therefore, it is not surprising that both display a positive correlation with *Igf2* transcription levels. This indicates that at least one is truly correlated with *Igf2* transcription. *p* values indicate that the correlation with *91H* RNA is more significant (*p* = 6.10^−5^) than that with *H19* RNA (*p* = 10^−3^). This result suggests that it is essentially the ectopic *91H* RNA up-regulates *Igf2* transcription in *trans*. Moreover, very large amounts of ectopic *H19* RNA, as observed in clones 4, 11 and 12ND (see Figure 4B), can counteract this effect. Indeed, by plotting *Igf2* transcription levels *versus* the ratio of *91H/H19* ectopic RNA levels, we observed a clear negative effect of ectopic *H19* RNA on *Igf2* transcription in these clones where the *91H/H19* ratio was inferior to 0.2 (Figure 6C, black diamonds). Finally, in all other clones, where *H19* RNA is much lower (in which the *91H/H19* ratio was superior to 0.2), the levels of the *H19* RNA do not display any significant effect on *Igf2* transcription levels (Figure 6C, white diamonds).

We thus propose that the relative sense/antisense ectopic *H19* RNA levels are able to control *Igf2 trans*-activation in our complementation assay.

### *Igf2 Trans*-activation by Ectopic *91H* RNA can Occur Without *H19* ICR Re-methylation

We then assessed whether *Igf2* up-regulation in transfected *H19* KO myoblasts is accompanied by re-methylation of the *H19* ICR. In addidtion, we also investigated the methylation levels of the other Differentially Methylated Regions (DMRs) of the locus. In order to determine DMR methylation levels at the *Igf2/H19* locus, we used digestions by methylation-sensitive restriction enzymes of DNA from untransfected and transfected *H19* KO myoblasts as well as control myoblasts (paternal heterozygous) (Figure S6). These experiments confirm that untransfected *H19* KO myoblasts are poorly methylated on the *H19* ICR (Figure S6A) and showed that *Igf2* DMR1 also becomes unmethylated (Figure S6B) while *Igf2* DMR2 remains highly methylated (Figure S6C). Low methylation levels are also found at the *H19* promoter (Figure S7A). However, the IgDMR at the *Dlk1/Gtl2* locus on chromosome 12 remains methylated (Figure S7B) indicating that the unmethylation observed at the *H19* ICR is not a general phenomenon, since it is not found at another imprinted locus. In transfected clones, the ICR (Figure S6A) and *Igf2* DMR1 (Figure S6B) show low DNA methylation levels while *Igf2* DMR2 remains largely methylated (Figure S6C). We conclude that ectopic *91H* and *H19* RNA expressions do not convincingly change DNA methylation patterns observed in untranfected *H19* KO myoblasts.

Finally, bisulfite-sequencing experiments confirmed that *H19* KO myoblasts are indeed very poorly methylated on the *H19* ICR (Figure S6E) and that the transfected *H19* KO myoblasts that displays the highest *Igf2* expression level (clone 4) is not re-methylated at the *H19* ICR (Figure S6F). Oppositely, the *H19* ICR is highly methylated in control myoblasts (+/− and C2C12 cells) (Figure S6D and S6G). These results clearly indicate that *Igf2 trans*-activation by ectopic *91H* RNA occurs without *H19* ICR re-methylation.

### *Igf2 Trans*-activation by Ectopic *91H* RNA Occurs Through Up-regulation of a Novel *Igf2* Promoter

**Figure 7:**
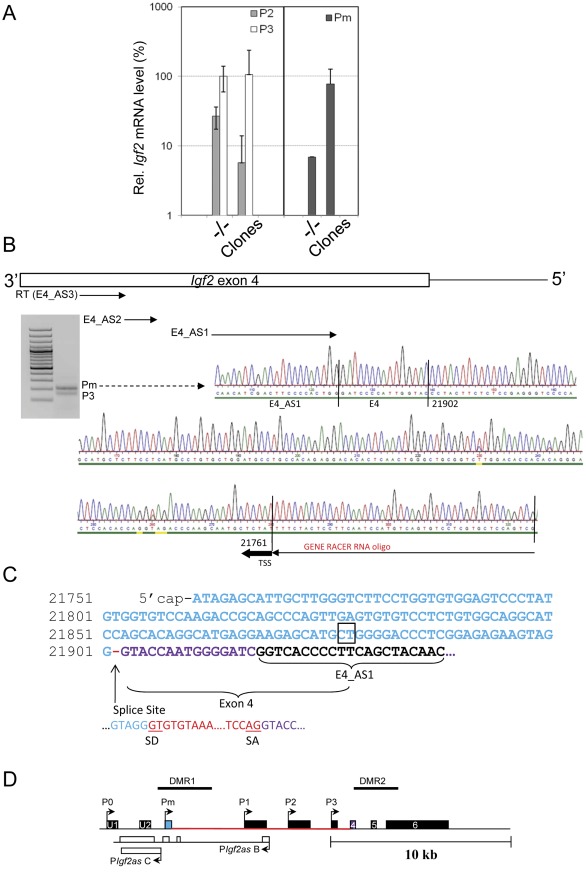
Characterization of a novel *Igf2* Pm transcript. (A) Comparison of *Igf2* transcripts produced from the P2/P3 (left panel) or Pm (right panel) promoters in untransfected (−/−) and transfected (“clones”) *H19* KO myoblasts. Transcript levels are given relative to the P3 transcript level in untransfected *H19* KO myoblasts (−/−) (100%). (B) Characterization of the TSS of the endogenous *Igf2* Pm transcript. 5′RACE experiment was performed on total capped RNA from transfected *H19* KO myoblasts (clone 4). The primer used for RT is the E4_AS3 and PCR reactions were performed using the Gene Racer primer and the E4_AS2 and then the E4_AS1 primers for nested PCR. The ethidium bromide staining of an agarose gel shows PCR products obtained from nested PCR amplifications. Sequencing of PCR product (Pm band) showed that it corresponds to a novel TSS of the *Igf2* gene (position chr7: 149,852,285 on mouse July 2007/ mm9 Assembly). The smaller band in the gel corresponds to the *Igf2* P3 transcript (data not shown). On the left is shown a 100bp-molecular weight ladder (C) The sequence of the novel *Igf2* Pm exon is given in blue and the novel splice site with exon 4 (purple) is indicated below. Intronic sequences are indicated in red. SD =  Splice Donor; SA = Splice Acceptor; The indicated CT dinucleotide corresponds to the splice acceptor site of an intron of a *Igf2* antisense RNA [58]. (D) Map of the *Igf2* gene showing the *Igf2* Pm promoter (blue rectangle). The sense and antisense *Igf2* exons are shown as black and white rectangles respectively. The first intron of the Pm *Igf2* mRNA is indicated in red.

**Figure 8:**
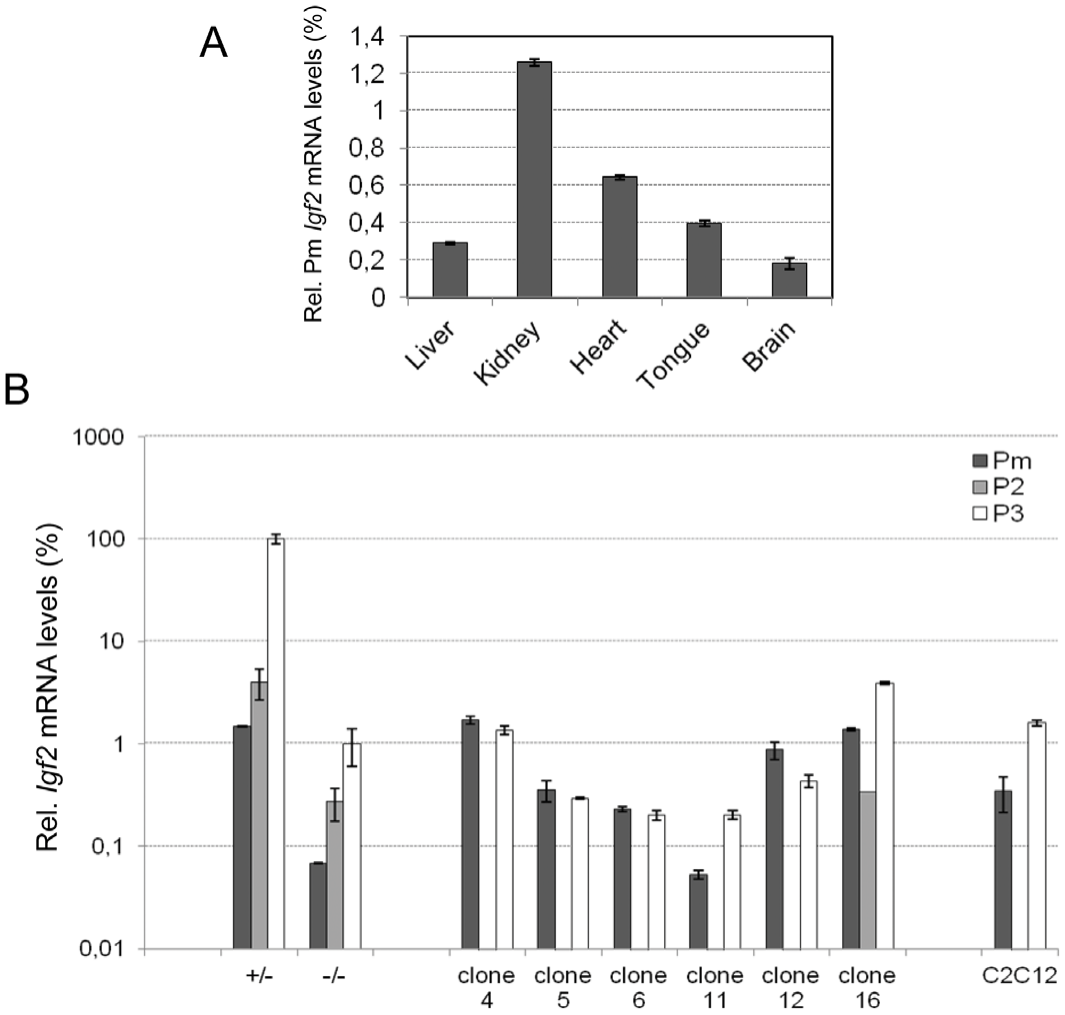
*Igf2* Pm mRNA levels in mouse tissues and *Igf2* promoter usage in transfected *H19*KO myoblasts. (A) Pm *Igf2* mRNA levels relative to total *Igf2* mRNA level (100%) in different mouse tissues. Total *Igf2* mRNA levels were determined by RT-qPCR using a PCR primer pair (Igf2exon6 PCR amplicon) located in the exon 6 common to all *Igf2* transcripts. (B) *Igf2* promoter usage in untransfected/transfected *H19* KO myoblasts. Relative mRNA levels (%) are calculated relative to the P3 *Igf2* transcript level in the control cells (+/−) (100%).

Since *Igf2 trans*-activation by ectopic *91H* RNA can occur without *H19* ICR re-methylation, we decided to investigate in more detail regulation of *Igf2* mRNA expression in transfected clones. Surprisingly, promoter usage analyses showed that the strongest P2/P3 *Igf2* promoters were not up-regulated in transfected *H19* KO myoblasts (Figure 7A, left panel). Since, the P0 and P1 promoters were not significantly expressed (data not shown), we hypothesized that *Igf2* up-regulation may occur by activation of an unknown *Igf2* promoter. To assess this possibility, we performed 5′ RACE experiments in clone 4 that displays the highest *Igf2* transcriptional activity (see Figure 5B). We identified a novel capped *Igf2* mRNA which contains a new exon which is 141nt long and is spliced to the exon 4 common to all known *Igf2* mRNAs (Figure 7B/C). This new transcript is initiated from a novel TSS located in the DMR1 (position chr7: 149,852,285 on mouse July 2007 mm9 Assembly). Using primers specific of the new *Igf2* exon, we determined the levels of this novel *Igf2* mRNA in different mouse tissues and showed that, while being poorly abundant, it is more expressed in mesodermic tissues (kidney, tongue and heart) than in liver or brain. We therefore called it “mesodermic” promoter (Pm) (Figure 8A).

We then analysed the Pm transcript in all our myoblastic cell lines. It turns out that this mRNA, like the other *Igf2* transcripts, was down-regulated in the *H19* KO myoblasts compared to control myoblasts (+/−) (Figure 8B). Remarkably, opposite to all the other *Igf2* transcripts, it was *trans*-activated in all transfected *H19* KO myoblasts except clone 11 which displays the lowest DMR2 methylation levels in addition to ICR hypomethylation (see Figure S6C and S6A respectively). Globally, the mean Pm *Igf2* mRNA level showed 10 fold up-regulation in transfected clones compared to untransfected *H19* KO myoblasts and reached P3 *Igf2* mRNA levels (Figure 7A, right panel). Therefore, we conclude that *Igf2 trans*-activation by ectopic *91H* RNA occurs essentially through up-regulation of the Pm *Igf2* promoter.

## Discussion

We recently contributed to show that, in the human, a large antisense *H19* transcript (*91H* RNA) regulates *Igf2* mRNA levels [34] whereas, in the mouse, the *H19* RNA is a negative *trans*-regulator of *Igf2* mRNA levels [33]. In the present work, we derived an *H19* KO myoblast cell line from mice carrying a deletion of the *H19* transcription unit (*H19* ^Δ*3*^) [36] in which the *Igf2* gene is repressed. Remarkably, loss of *Igf2* transcription in *H19* KO myoblasts correlates with a loss of *H19* ICR methylation. It is therefore very likely that the CTCF protein binds to the ICR on both parental alleles leading to an almost complete insulation of the regular P2/P3 *Igf2* promoters from the enhancers. Using a genetic complementation approach (reintroduction of the *H19* sequence in *H19* KO myoblasts), we investigated steady-state levels and halves-lives of ectopic *91H* and *H19* RNAs, as well as endogenous *Igf2* transcriptional activity, and we show (i) that strong ectopic expression of antisense *H19* transcripts synthesized from the enhancer 2 region can release *Igf2* silencing in mouse myoblasts (ii) that this *Igf2* reactivation takes place at the transcriptional level by targeting a previously unknown *Igf2* promoter and (iii) that a large amount of ectopic *H19* RNA can counteract *Igf2 trans*-activation by ectopic *91H* RNA. Strikingly, we show that *trans*-activation of this novel Pm *Igf2* promoter occurs without *H19* ICR re-methylation indicating that this promoter is able to by-pass the insulator function of the unmethylated ICR. It thus remains possible that Pm activity also occurs on the maternal allele. This effect may potentially rely on the activity of the DMR2 that remains largely methylated in our experimental system (Figure S6C) and is known in mouse to favour *Igf2* transcription on the methylated paternal allele [39]. This possibility would be reminiscent to some human pancreatic tumors like insulinomas where *Igf2* DMR2 is hypermethylated while the *H19* ICR is monoallelically methylated and where *Igf2* becomes also expressed from the unmethylated maternal allele (loss of imprinting) [40].

**Figure 9:**
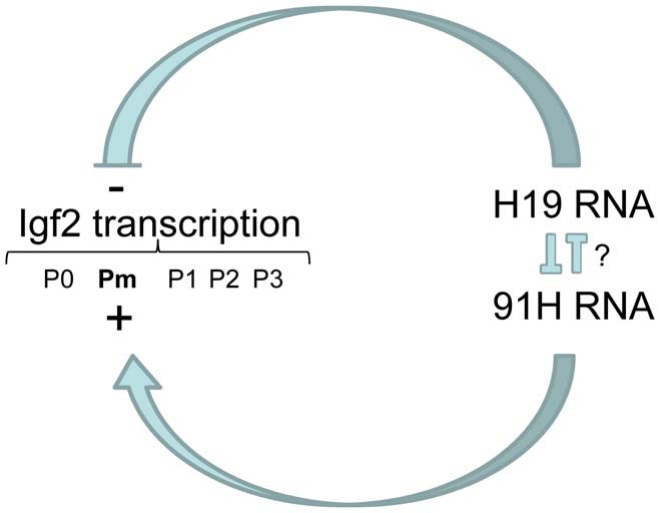
Model of regulation of *Igf2* transcription by *91H* and *H19* RNAs in myoblastic cells. This model is based on the three critical parameters that we have quantified in this work: the *Igf2* transcription levels, the *91H* and *H19* steady state RNA levels. *91H* and *H19* RNAs are direct and/or indirect antagonists riboregulators of *Igf2* transcription. H19 appears as having a negative effect on *Igf2* transcription while *91H* RNA has an opposite effect. Interestingly, *91H* RNA stimulates a new *Igf2* promoter (Pm) located within the *Igf2* Differentially Methylated Region 1 (DMR1).

Altogether, our inactivation/complementation approach, in conjunction with other recent findings [33], [34], reveals that the mouse *H19* antisense RNA favours *Igf2* transcription and activates the *Igf2* Pm promoter while large amounts of the *H19* transcript counteract this effect, suggesting that these two transcripts are antagonist *trans* riboregulators (Figure 9). Therefore, in cells like the C2C12 myoblasts where we observe very low amounts of *91H* RNA and large amounts of *H19* RNA, one can expect that the endogenous *H19* RNA exerts a strong *Igf2* transcriptional repression at least on the Pm promoter.

Our experiments also agrees with the pioneer work by Wilkin *et al*. which suggested that, in human, a partial *H19* cDNA construct could activate *IGF2* when expressed in the antisense orientation while *H19* RNA can repress transcription from the *IGF2 P3* promoter [37]. However, at that time, the endogenous antisense *H19* RNA was unknown and its effect in this work remained enigmatic.

Our results reveal a functional relationship between *H19* and *91H* RNAs. Consequently, depending on the cell context, the functional relevance of the *H19* transcriptional unit for *Igf2* gene control will depend on the relative expression levels of the sense and antisense *H19* transcripts. This finding is particularly relevant for a better understanding of the conflicting data obtained for *H19* gene expression in cancer cells and tumours. Indeed, *91H* RNA levels should be taken into consideration as this transcript is a good marker of tumourigenesis in breast cancer cells [34]. In summary, the *91H* RNA could be assumed to be oncogenic by favouring *Igf2* transcription while *H19*, which counteracts this effect, would act as a tumour suppressor [23]. Consistently, normal breast tissues display high *H19* and very low *91H* RNA levels, while the opposite is observed in cancerous breast tissues [34]. Interestingly, the effect of the *91H* RNA on *Igf2* derepression observed here in complementation studies, may explain the *Igf2* derepression occurring in many tumours where *91H* RNA was found to accumulate while the *H19* gene is maintained in a repressed state [34]. One can note that an *H19* antisense transcript called *H19* opposite tumor suppressor (HOTS) was recently found in human [41]. It extends from 2.8 kb downstream of *H19* to 1 kb upstream and is encoding a nucleolar protein which is not conserved in the mouse. An evolutionarily conserved microRNA miR675 has been also described in the *H19* exon 1 [42], [43]. Recently, this *H19*-derived miR-675 was shown to regulate tumor suppressor RB in human colorectal cancer favoring its progression [44]. An interesting possibility is that this miRNA may also be directly involved in controlling levels of *91H* RNA. An open question is why, *in vivo*, so much *H19* RNA would be required to produce this miRNA and to control such small amounts of *91H* RNA? This may be due to the fact that the *H19* RNA is mainly cytoplasmic while the *91H* transcript is nuclear [34]. Therefore, only a small sub-set of nuclear *H19* RNA may be involved in this process. Furthermore, the miRNA production does not appear to significantly affect *H19* RNA levels and therefore this process should not interfere with other functions that the *H19* RNA may have in the cytoplasm where it is known to localize with the polysomes [38], [45]. Alternatively, the opposite effects of the *91H* and *H19* RNAs on *Igf2* transcription may occur through more indirect mechanisms involving for example the *Igf2* DMRs.

Here, upon isolation of *H19* KO mouse myoblasts, the *Igf2* gene expression was strongly decreased after passages in cell culture probably due to the observed loss of *H19* ICR DNA methylation. It is formally possible that methylation levels have changed as a consequence of cell culture. Alternatively, this may also result from the deletion of the *H19* transcriptional unit. In the mouse mesoderm-derived tissues, and more particularly in the postnatal muscle, maternal inheritance of the *H19* ^Δ*3*^ deletion is known to lead to loss of *Igf2* imprinting and re-expression of this gene from the maternal allele [23], [36]. In the physiological context of this tissue, the presence of other cell types, such as for example satellite cells, may strongly contribute to maintain normal *Igf2* levels by signalling through intercellular pathways which may control myoblast cell differentiation [46]. In the present study, we reactivated *Igf2* transcription in *H19* KO myoblasts by ectopic *91H* RNA expression without *H19* ICR re-methylation. Although we could not investigate whether this reactivation is monoallelic or biallelic, both parental alleles are largely unmethylated since methylation levels are very low (Figure S6) indicating that *Igf2* reactivation could occur on both parental alleles.

It now would be of interest to modify in the animal the *91H* RNA levels independently of *H19* RNA levels, as performed above in the myoblast *H19* KO cell line. Unfortunately, such an experiment is tricky to do *in vivo* since both RNAs possess identical expression patterns and are both produced from the maternal allele. Furthermore, the investigations should be performed at the transcriptional level (nuclear run-on assays) to exclude any potential post-transcriptional effects. Finally, a transgenic line that would display high ectopic *91H* RNA but low ectopic *H19* RNA has not yet been produced. Such a transgenic mouse strain would be important to confirm the results obtained here in myoblast cell lines. However, *91H* RNA expression should be altered in the *minute* mouse mutant. Indeed, Davis et al. [47] have shown that the *minute* (*Mnt*) mutation is an inversion that disrupts a candidate region for muscle specific enhancers and, in mesodermic tissues, *91H* RNA is initiated downstream of the endodermic enhancers, most probably in the sequence inverted in *minute* mice. However, we cannot rule out that the *H19* antisense RNA expression may persist in *Mnt* mice by activation of some propitious transcription start sites. Therefore, it may be interesting to investigate *91H* and *Igf2* Pm transcript levels in this mouse mutant.

Since we previously demonstrated that the *91H* RNA acts in *trans* on *Igf2* mRNA levels in human cells, one could hypothesize that this RNA may act exclusively at the post-transcriptional level. The present work clearly demonstrates that this is not the case, and that the *91H* RNA augments *Igf2* expression by acting at the transcriptional level. This finding raises the question about the mechanisms involved in such a regulation. *Igf2* transcriptional regulation is known to be controlled through long-range interactions between regulatory elements, such as the Differentially Methylated Regions (DMRs) and the enhancers located downstream of *H19* [18], [48], [49]. We could therefore propose that the *91H* RNA can up-regulate tissue-specific *Igf2* transcription by contributing, directly or indirectly, to higher-order chromatin architecture of this locus. For example, it may favour interactions between the *Igf2* gene and specific enhancers since our experiments show that, in myoblastic cells, the *91H* RNA can reactivate the Pm *Igf2* promoter which is used in mesodermic tissues. Alternatively, the *91H* RNA could also titrate factors such as transcriptional repressors, targeting *Igf2* as well as some other genes of the Imprinted Gene Network (IGN) [32]. Finally, the *91H* RNA is produced in liver from the endodermic enhancers (Figure 2) that themselves control the *H19* expression levels in *cis* [50]. It is also able to act in *trans* to control *Igf2* transcription (Figure 5) and to up-regulate the Pm transcript (Figure 8). Therefore, this lncRNA appears as a novel important player for co-regulation of genes at the *Igf2/H19* locus.

## Materials and Methods

### Ethics Statement

All experimental designs and procedures are in agreement with the guidelines of the animal ethics committee of the French “Ministère de l’Agriculture”. Our animal unit has been registered at the departmental office for population protections (*Direction départementale de la protection des populations*) at the “Hérault préfecture” (Agreement N°E34-172-16). All the experimental protocols (mouse dissections) have been specifically approved by the inspector in charge of the veterinary public heath from the same office at the “Hérault préfecture” (Agreement N°34-31).

### Isolation of *H19* KO Myoblasts

Primary cultures were prepared from the thigh muscles of H19^Δ3^ mice as previously described [51]. Primary cells (*H19* KO and control paternal heterozygous myoblasts) were serially passaged for analysis. Using qPCR on genomic DNA we checked that, as expected, the isolated *H19* KO myoblasts were devoid of the *H19* transcription unit and that the *Igf2* gene was in an identical copy number in *H19* KO myoblasts as in C2C12 myoblast cells, suggesting that no aberrant loss or duplication of chromosome 7 occurred in the *H19* KO myoblast cell line (data not shown).

### Cell Culture and Transfections

*H19* KO (−/−) and control paternal heterozygous (+/−) myoblasts were cultured in DMEM/MCDB 1∶1, containing 20% FCS and 2% Ultroser (Gibco). Cells were differentiated into myotubes upon 3 days of serum starvation. The 16 kb BamHI-BamHI fragment corresponding to the *H19* gene locus (Figure 1) was cloned into the *Not I* site of the pBluescript plasmid using appropriate linkers. The construct was digested with *Not I* and the insert was gel-purified before being co-transfected with a hygromicin-resistance plasmid into *H19* KO myoblast cells using lipofectamin (Gibco) according to the recommendations of the manufacturer. Actinomycin D at a final concentration of 5 µg/ml was added to the cell culture medium for the times indicated in figure legends.

### RNA Isolation, Northern-blot and RT-qPCR Analyses

Total RNA was isolated from mouse tissue samples or from myoblastic cells by the guanidinium thiocyanate procedure as previously described [38]. Non-polyadenylated RNAs were prepared by using the PolyA Tract mRNA isolation system III® (Promega). The *Igf2* and H19 RNAs were analysed in Northern-blots as previously described [52]. Reverse transcriptions and real-time quantitative PCRs were performed as previously described [34], [53] using a qPCR mix described in Lutftalla *et al.* [54] with some modifications given in Court *et al.* [55]. The *Igf2* steady-state mRNA levels were quantified using a PCR amplicon which targeted the messenger RNA (for primer sequences see Table S1). The ectopic *H19* RNA levels were quantified using primers located at *H19* exon-exon junctions while the ectopic *91H* transcript was quantified either in the intergenic region between the endodermic enhancers and the *H19* gene (mC’, mD, mE, mF and mJ PCR amplicons) or within the *H19* introns (mI1, mI2 and mI3 PCR amplicons) (Figure 1 and Table S1). Indeed, since the levels and the halve-lives of the RNAs quantified by the intronic PCR amplicons are similar to those quantified by the mC’ PCR amplicon, we assume that the *H19* intron sequences essentially account for the ectopic *91H* RNA in transfected *H19* KO myoblasts (Figure S5). Throughout this work RNA levels determined by RT-qPCR were expressed relative to *Gapdh* mRNA levels. *Igf2* promoter usage was assessed by quantifying transcripts on each promoter-specific exon (first exons).

### 5′RACE

Rapid Amplification of 5′ complementary DNA Ends (5′RACE) was performed on non-polyadenylated d7 mouse liver RNAs (endogenous *91H* RNA) or transfected *H19* KO myoblast RNAs (clone 4) (ectopic *91H* RNA and endogenous *Igf2* Pm transcript) according to manufacturer’s instructions (GeneRacer® Kit from *Invitrogen* ref. L1502). RT and nested PCR Primer sequences are given in Table S1.

### Transgene Copy-number Determination

Transgene copy-numbers were determined by qPCR relative to the endogenous *Igf2* gene.

### Nuclear Run-on

Isolation of nuclei and nuclear run-on experiments were performed as previously described [38], [53].

### DNA Methylation Analyses

Each sample was digested by the StyI restriction enzyme (20 units) to eliminate potential PCR bias due to the reduced accessibility of primers on undigested genomic DNA [56]. For *H19* ICR methylation analyses (CTCF site 2), half of each samples was then additionally digested by the BceAI methylation-sensitive enzyme (4 Units/reaction) and qPCR quantifications were performed on BceAI-digested and undigested fractions after normalization against a loading control (242C19 primer pair). A similar approach was followed using the methylation-dependent McrBC enzyme to determine methylation levels in the Ig-DMR (*Dlk1/Gtl2* locus on mouse chromosome 12) [57] or methylation-sensitive enzymes (NaeI for *Igf2* DMR1 and HpaII for *Igf2* DMR2). Methylation levels of the CpG residues studied for the *H19* ICR and *Igf2* DMRs are known to be representative of DNA methylation levels of the whole DMRs [52]. Primer sequences are available in Table S1.

### Bisulfite Treatments

Genomic DNA was prepared from myoblastic cells and conversion with sodium bisulfite was performed with the Epitect® kit (Qiagen) following the manufacturer’s instructions. PCR fragments were cloned using a PCR cloning Kit from Qiagen. Clones with strictly identical patterns of conversion were removed from the results (since they are likely to represent identical molecules). We used the MethPrimer software to design primers on bisulfite treated DNA. Primer sequences are available in Table S1.

## Supporting Information

Figure S1 Differentiation of *H19* KO myoblast cells. The figure shows pictures of the *H19* KO myoblasts under the optical microscope (20× enhancement) during the myogenic differentiation process (ND =  undifferentiated; d1, d2 and d3 correspond to 1, 2 or 3 days of differentiation). The transcriptional levels of the *myogenin*, a myogenic marker, are up-regulated during differentiation of *H19* KO myoblast cells with the same amplitude (6–7 fold) as observed in C2C12 myoblasts (data not shown).
(TIF)

Figure S2 Quantifications of the intact or truncated endogenous *91H* RNA levels (A) and of *Igf2* mRNA (B) relative to *gapdh* mRNA levels in myoblast cell lines. (A) Comparison between the intact (black bars) and truncated (grey bars) endogenous *91H* RNA levels determined by RT-qPCR in C2C12 myoblasts and *H19* KO myoblasts respectively. (B) Quantification of *Igf2* mRNA levels during differentiation of *H19* KO myoblasts (late passage cells) (ND =  undifferentiated; D =  differentiated). One can note that, as observed for the endogenous truncated *91H* RNA (Figure S2A), the low *Igf2* levels observed in *H19* KO myoblasts were strongly up-regulated (by at least 20-fold) during myogenic differentiation (Figure S2B). This suggests that the *H19* transcription unit is dispensable to *Igf2* up-regulation processes observed during myogenic differentiation.
(TIF)

Figure S3 Characterisation of TSS of the ectopic mouse *91H* RNAs. 5′RACE experiments were performed on total capped RNA from transfected *H19* KO myoblasts (clone 4). (A) Map of the enhancer region showing the primers used for RT and PCR reactions. The RT was initiated from the forward primer of PCRa. (B) Ethidium bromide staining of an agarose gel showing PCRs product obtained from amplifications as indicated in Figure 2B) (MW: Molecular Weight). Sequencing of PCRa product showed that this band corresponds essentially to unspecific amplification while PCRb correspond to the major TSS of the *91H* RNA (position chr7:149,755,206 or chr7:149,755,207 on mouse July 2007/ mm9 Assembly) and PCRc contains two minor TSS initiated within the endodermic enhancer 2 sequence upstream of the major TSS. These minor TSS could be identified in this experiment probably because ectopic *91H* RNA is overexpressed compared to its endogenous counterpart. (C) The sequence of the endodermic enhancer 2 is indicated in bold. The positions of the minor and major TSS are indicated by black arrows. Due to the presence identical nucleotidic sequences at the end of the GeneRacer RNA oligonucleotide primer and at the TSS, the exact position of the major and one minor TSS remain ambiguous.
(TIF)

Figure S4 Endogenous *vs* ectopic *91H/H19* RNA half-lives. (A) Stability of the endogenous *91H* and *H19* RNAs in C2C12 myoblasts. C2C12 myoblast cells were treated with Actinomycin D and relative RNA levels were determined by real time RT-qPCR at the indicated times (in hours). Data were normalized to *Gapdh* expression levels. *H19* (*H19* RNA PCR amplicon), *91H* (RT-qPCR quantifications with the mC’ PCR amplicon) and *H19* precursor (intron 2, mI2 PCR amplicon) RNA levels are shown. Note that the half-life of the *91H* RNA (middle panel) is similar to that of an unspliced *H19* precursor RNA (right panel). (B) Stability of the ectopic *91H* and *H19* RNAs were determined in transfected *H19* KO myoblasts using the same PCR amplicons as above. The whole hygromycin-resistant transfected *H19* KO myoblast cell population was treated with Actinomycin D as described above and the ectopic *H19* and ectopic *91H* RNA levels were quantified as indicated above. Note that the ectopic *91H* RNA appears to be more stable than the endogenous *91H* transcript in C2C12 cells (compare Figure S4A with Figure S4B). This may be due to the 1000-overexpression of the ectopic *91H* RNA found in transfected *H19* KO myoblasts relative to the endogenous levels observed in C2C12 cells (Figure 4B, compare right and left panels). Since, in transfected KO myoblasts, the ectopic *91H* RNA is found in similar amounts as the ectopic *H19* RNA (Figure 4B, left panel) despite its low stability (Figure S4B), we should conclude that ectopic *91H* transcription is much higher than that of the ectopic *H19*.
(TIF)

Figure S5 RT-qPCR quantifications of *91H* RNAs. Note that, in C2C12 cells, quantifications by the mI1-mI3 PCR amplicons (blue bars) account for the endogenous *H19* precursor RNA level but not the endogenous *91H* transcript which is much lower as shown using the mC’ PCR amplicon (red bar). In the opposite, in transfected *H19* KO myoblasts, quantifications using the mI1-mI3 PCR amplicons, as well as with the mC’ PCR amplicon, account for the ectopic *91H* RNA level which is very high. The *91H* RNA levels shown in Figure 4B corresponds to the mean of quantifications using mC’ and mI1-mI3 PCR amplicons.
(TIF)

Figure S6 DNA methylation patterns of *H19* ICR and *Igf2* DMRs. Methylation patterns were analysed in control (+/−) and *H19* KO (−/−) myoblasts after 40 passages and in transfected clones, 3 passages after clonal isolation. The methylation pattern of the *H19* ICR (A), the *Igf2* DMR1 (B) and *Igf2* DMR2 (C) were estimated by digestion of the genomic DNA with methylation-sensitive restriction enzymes (BceAI, NaeI and HpaII for ICR, DMR1 and DMR2 respectively) and quantifications by qPCR. Noteworthy, this BceAI site encompasses CpG dinucleotides from CTCF site 2 of the *H19* ICR. Error bars represent s.e.m. of quantifications performed on at least two independent digestions. Methylation patterns of the *H19* ICR around CTCF site 2 was determined by bisulfite sequencing in control (+/−) (D), *H19* KO (E), clone 4 (F) and C2C12 (G) myoblasts. Black and white circles indicate methylated and unmethylated CpGs respectively.
(TIF)

Figure S7 Methylation patterns of *H19* promoter and IgDMR (*Dlk1/Gtl2* locus on mouse chromosome 12). DNA methylation patterns were analysed in control (+/−) and *H19* KO (−/−) myoblasts after 40 passages and in the transfected clone 4 (3 passages after clonal isolation) as well as in C2C12 cells. The methylation pattern of the *H19* promoter (A) and the IgDMR (B) were estimated by digestion of the genomic DNA with methylation-sensitive/dependent restriction enzymes (HpaII and McrBC for *H19* promoter and IgDMR respectively). Error bars represent s.e.m. of quantifications performed on at least two independent digestions.
(TIF)

Table S1 Sequences of the qPCR primers used in the present work. Primers indicated in bold were also used in RT reactions. For further details, also see Results and Materials & Methods sections.
(DOC)
