# Supplementary figures and images for: H19 Antisense RNA Can Up-Regulate Igf2 Transcription by Activation of a Novel Promoter in Mouse Myoblasts
Source: PLoS One. 2012 May 25;7(5):e37923. doi: 10.1371/journal.pone.0037923 (PMC3360672; doi:10.1371/journal.pone.0037923)

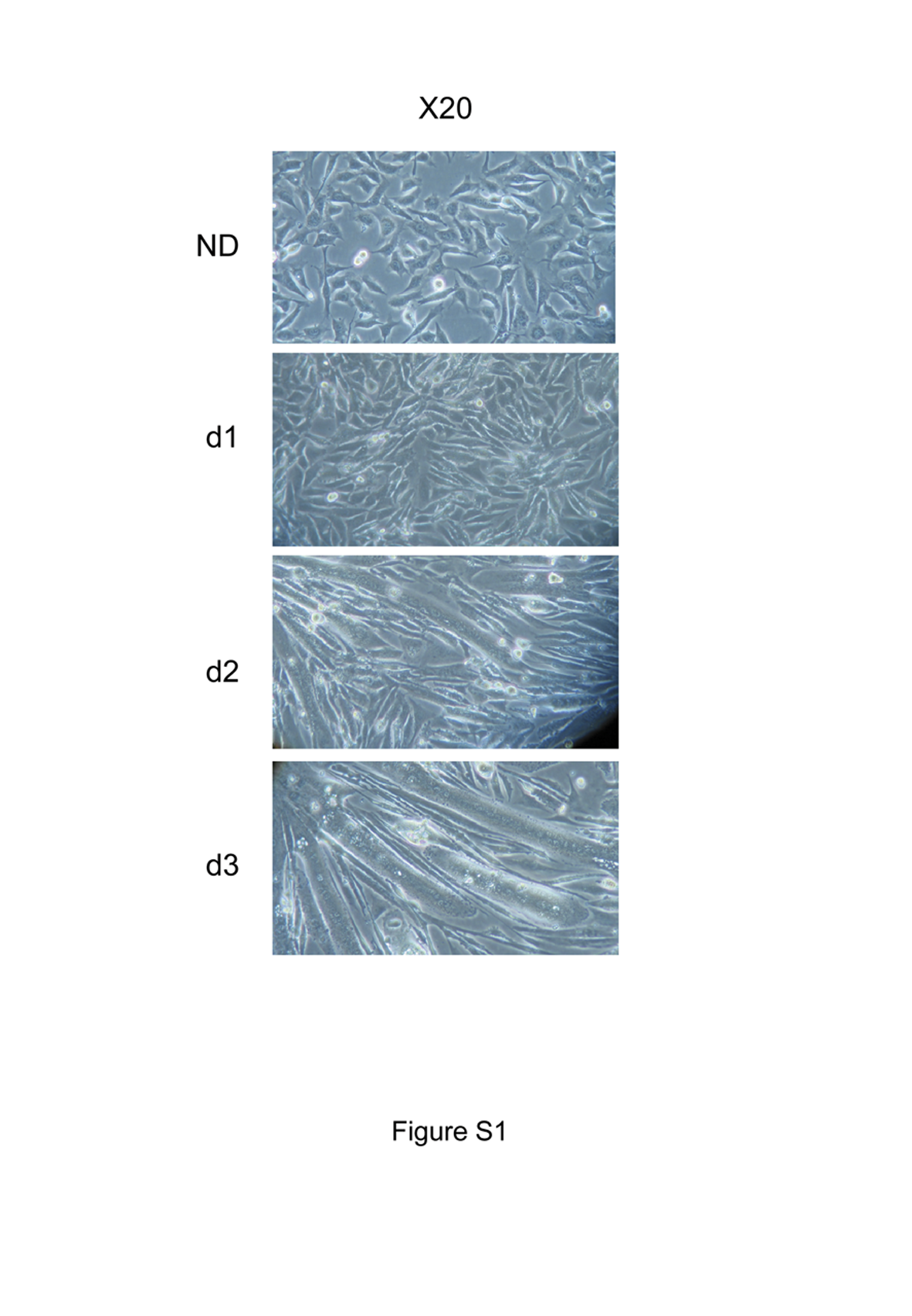

Supplement: Figure S1 — Differentiation of H19 KO myoblast cells. The figure shows pictures of the H19 KO myoblasts under the optical microscope (20× enhancement) during the myogenic differentiation process (ND = undifferentiated; d1, d2 and d3 correspond to 1, 2 or 3 days of differentiation). The transcriptional levels of the myogenin, a myogenic marker, are up-regulated during differentiation of H19 KO myoblast cells with the same amplitude (6–7 fold) as observed in C2C12 myoblasts (data not shown). (TIF) [file pone.0037923.s001.tif]

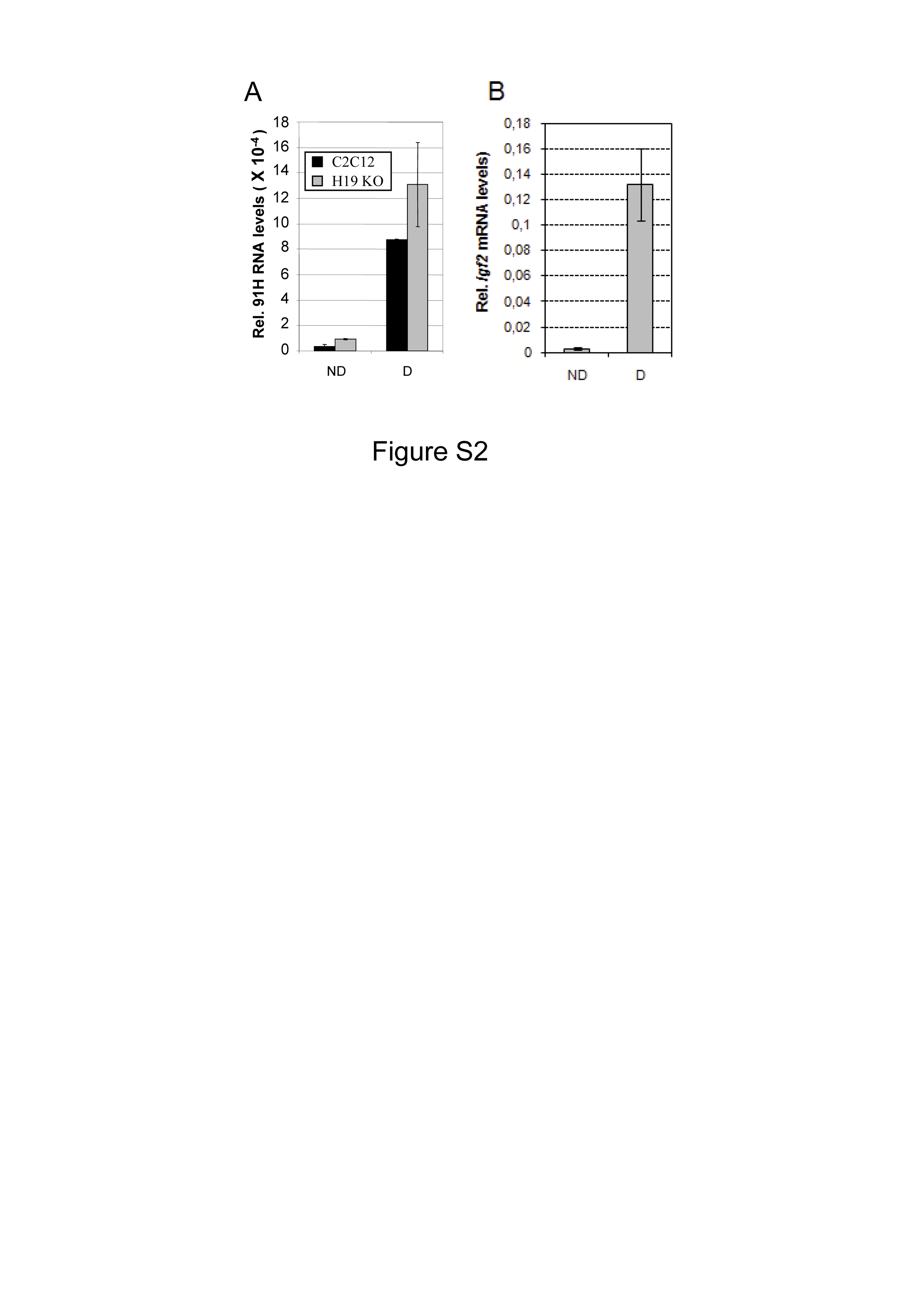

Supplement: Figure S2 — Quantifications of the intact or truncated endogenous 91H RNA levels (A) and of Igf2 mRNA (B) relative to gapdh mRNA levels in myoblast cell lines. (A) Comparison between the intact (black bars) and truncated (grey bars) endogenous 91H RNA levels determined by RT-qPCR in C2C12 myoblasts and H19 KO myoblasts respectively. (B) Quantification of Igf2 mRNA levels during differentiation of H19 KO myoblasts (late passage cells) (ND = undifferentiated; D = differentiated). One can note that, as observed for the endogenous truncated 91H RNA (Figure S2A), the low Igf2 levels observed in H19 KO myoblasts were strongly up-regulated (by at least 20-fold) during myogenic differentiation (Figure S2B). This suggests that the H19 transcription unit is dispensable to Igf2 up-regulation processes observed during myogenic differentiation. (TIF) [file pone.0037923.s002.tif]

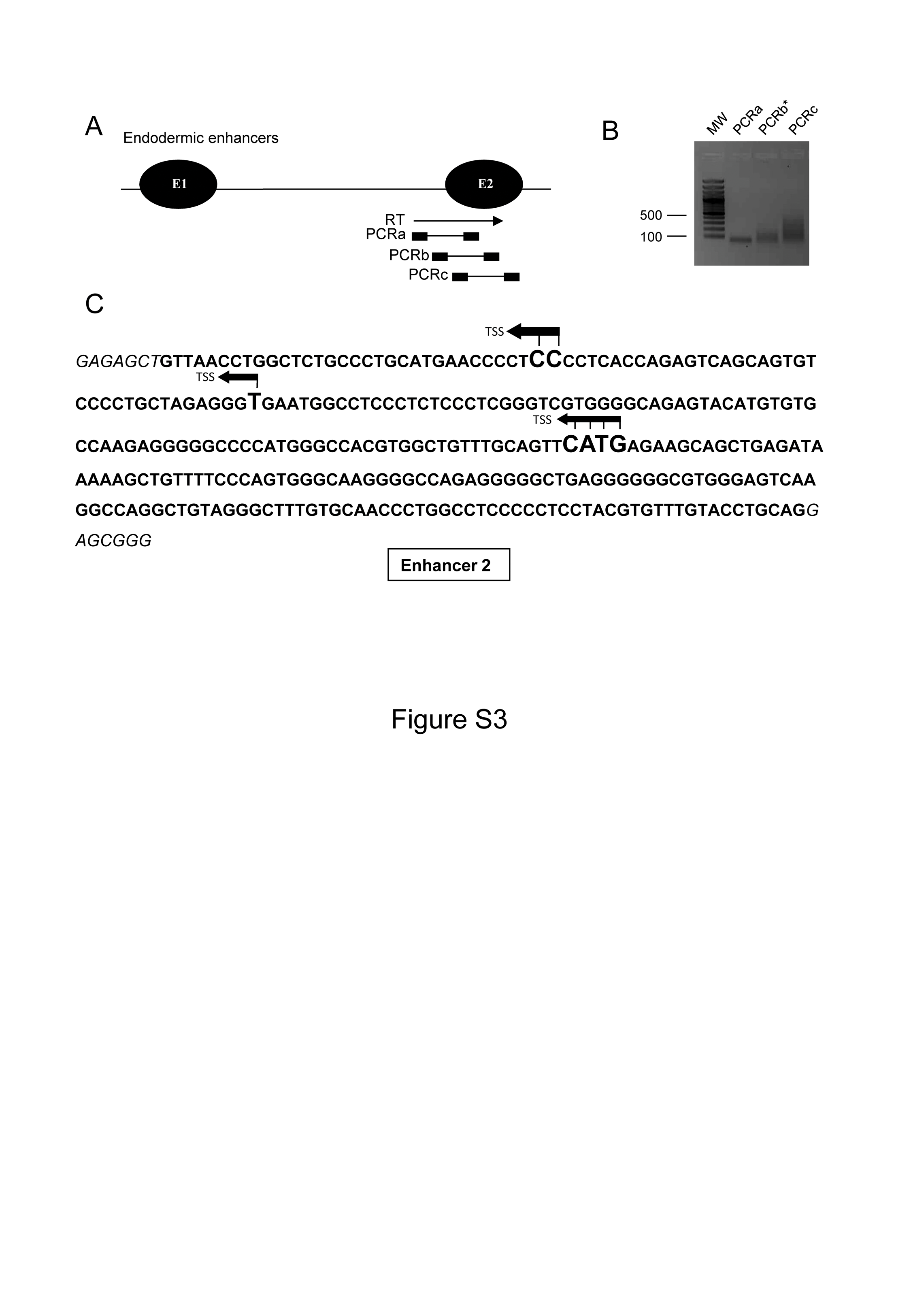

Supplement: Figure S3 — Characterisation of TSS of the ectopic mouse 91H RNAs. 5′RACE experiments were performed on total capped RNA from transfected H19 KO myoblasts (clone 4). (A) Map of the enhancer region showing the primers used for RT and PCR reactions. The RT was initiated from the forward primer of PCRa. (B) Ethidium bromide staining of an agarose gel showing PCRs product obtained from amplifications as indicated in Figure 2B) (MW: Molecular Weight). Sequencing of PCRa product showed that this band corresponds essentially to unspecific amplification while PCRb correspond to the major TSS of the 91H RNA (position chr7:149,755,206 or chr7:149,755,207 on mouse July 2007/ mm9 Assembly) and PCRc contains two minor TSS initiated within the endodermic enhancer 2 sequence upstream of the major TSS. These minor TSS could be identified in this experiment probably because ectopic 91H RNA is overexpressed compared to its endogenous counterpart. (C) The sequence of the endodermic enhancer 2 is indicated in bold. The positions of the minor and major TSS are indicated by black arrows. Due to the presence identical nucleotidic sequences at the end of the GeneRacer RNA oligonucleotide primer and at the TSS, the exact position of the major and one minor TSS remain ambiguous. (TIF) [file pone.0037923.s003.tif]

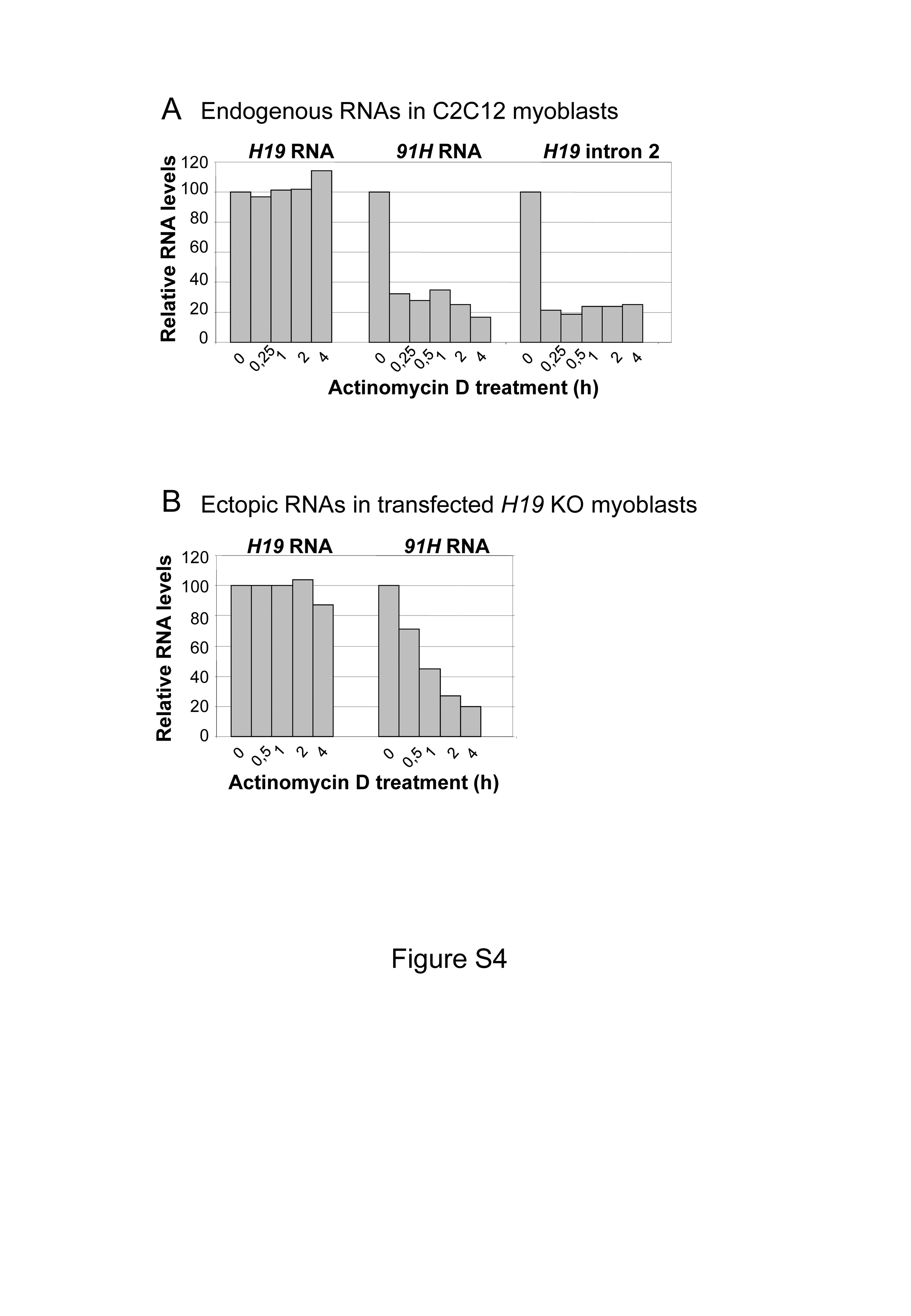

Supplement: Figure S4 — Endogenous vs ectopic 91H/H19 RNA half-lives. (A) Stability of the endogenous 91H and H19 RNAs in C2C12 myoblasts. C2C12 myoblast cells were treated with Actinomycin D and relative RNA levels were determined by real time RT-qPCR at the indicated times (in hours). Data were normalized to Gapdh expression levels. H19 (H19 RNA PCR amplicon), 91H (RT-qPCR quantifications with the mC’ PCR amplicon) and H19 precursor (intron 2, mI2 PCR amplicon) RNA levels are shown. Note that the half-life of the 91H RNA (middle panel) is similar to that of an unspliced H19 precursor RNA (right panel). (B) Stability of the ectopic 91H and H19 RNAs were determined in transfected H19 KO myoblasts using the same PCR amplicons as above. The whole hygromycin-resistant transfected H19 KO myoblast cell population was treated with Actinomycin D as described above and the ectopic H19 and ectopic 91H RNA levels were quantified as indicated above. Note that the ectopic 91H RNA appears to be more stable than the endogenous 91H transcript in C2C12 cells (compare Figure S4A with Figure S4B). This may be due to the 1000-overexpression of the ectopic 91H RNA found in transfected H19 KO myoblasts relative to the endogenous levels observed in C2C12 cells (Figure 4B, compare right and left panels). Since, in transfected KO myoblasts, the ectopic 91H RNA is found in similar amounts as the ectopic H19 RNA (Figure 4B, left panel) despite its low stability (Figure S4B), we should conclude that ectopic 91H transcription is much higher than that of the ectopic H19. (TIF) [file pone.0037923.s004.tif]

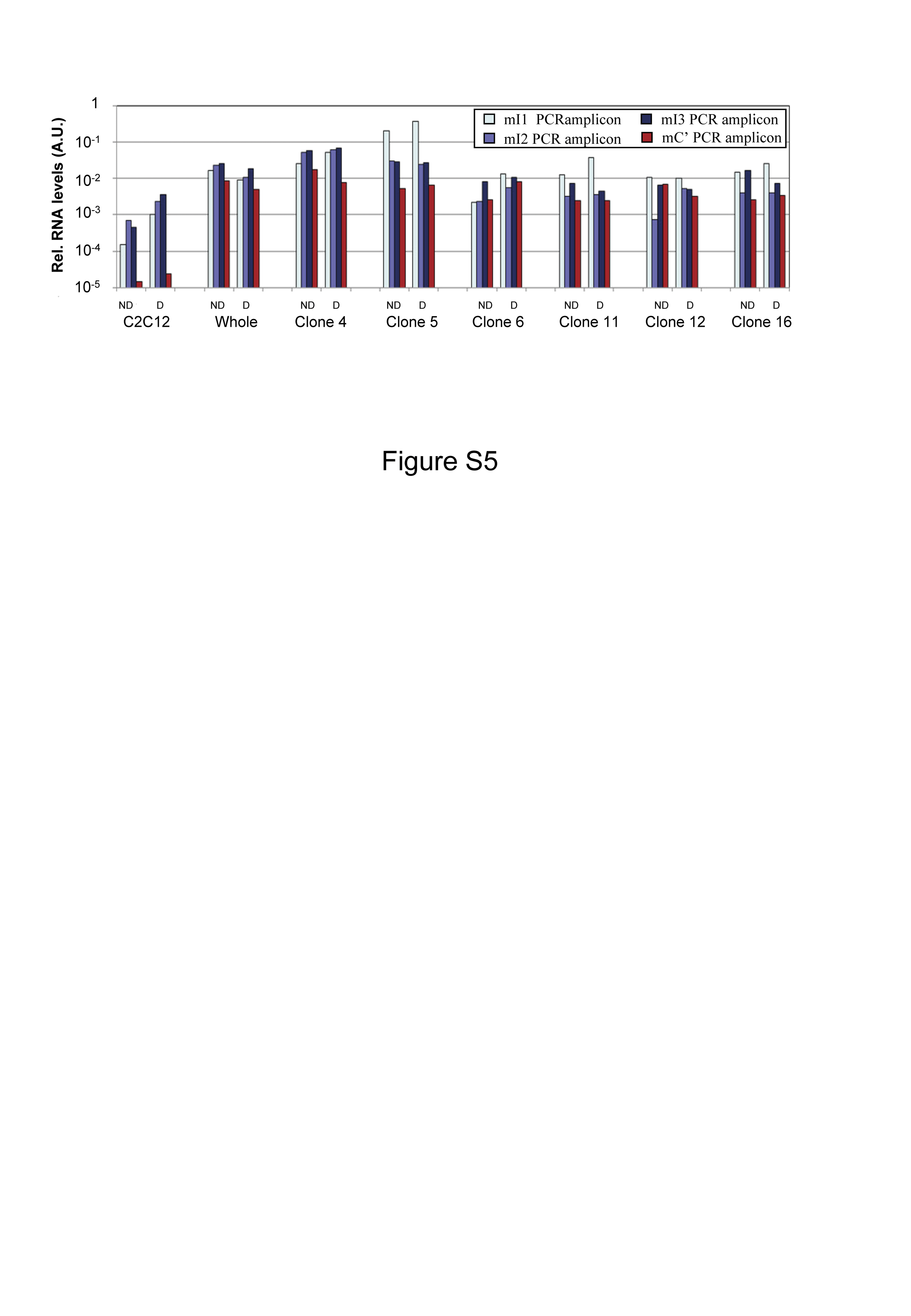

Supplement: Figure S5 — RT-qPCR quantifications of 91H RNAs. Note that, in C2C12 cells, quantifications by the mI1-mI3 PCR amplicons (blue bars) account for the endogenous H19 precursor RNA level but not the endogenous 91H transcript which is much lower as shown using the mC’ PCR amplicon (red bar). In the opposite, in transfected H19 KO myoblasts, quantifications using the mI1-mI3 PCR amplicons, as well as with the mC’ PCR amplicon, account for the ectopic 91H RNA level which is very high. The 91H RNA levels shown in Figure 4B corresponds to the mean of quantifications using mC’ and mI1-mI3 PCR amplicons. (TIF) [file pone.0037923.s005.tif]

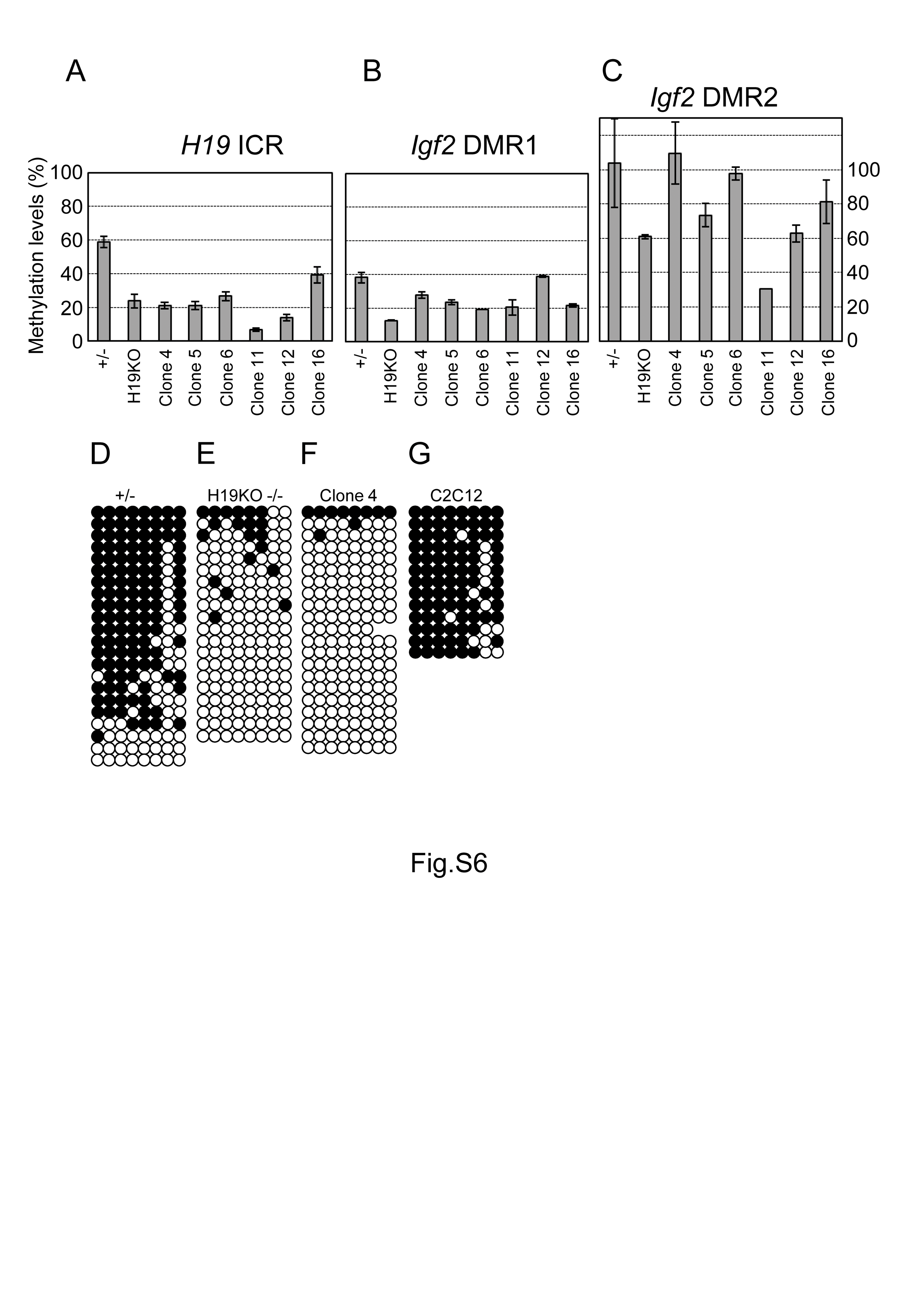

Supplement: Figure S6 — DNA methylation patterns of H19 ICR and Igf2 DMRs. Methylation patterns were analysed in control (+/−) and H19 KO (−/−) myoblasts after 40 passages and in transfected clones, 3 passages after clonal isolation. The methylation pattern of the H19 ICR (A), the Igf2 DMR1 (B) and Igf2 DMR2 (C) were estimated by digestion of the genomic DNA with methylation-sensitive restriction enzymes (BceAI, NaeI and HpaII for ICR, DMR1 and DMR2 respectively) and quantifications by qPCR. Noteworthy, this BceAI site encompasses CpG dinucleotides from CTCF site 2 of the H19 ICR. Error bars represent s.e.m. of quantifications performed on at least two independent digestions. Methylation patterns of the H19 ICR around CTCF site 2 was determined by bisulfite sequencing in control (+/−) (D), H19 KO (E), clone 4 (F) and C2C12 (G) myoblasts. Black and white circles indicate methylated and unmethylated CpGs respectively. (TIF) [file pone.0037923.s006.tif]

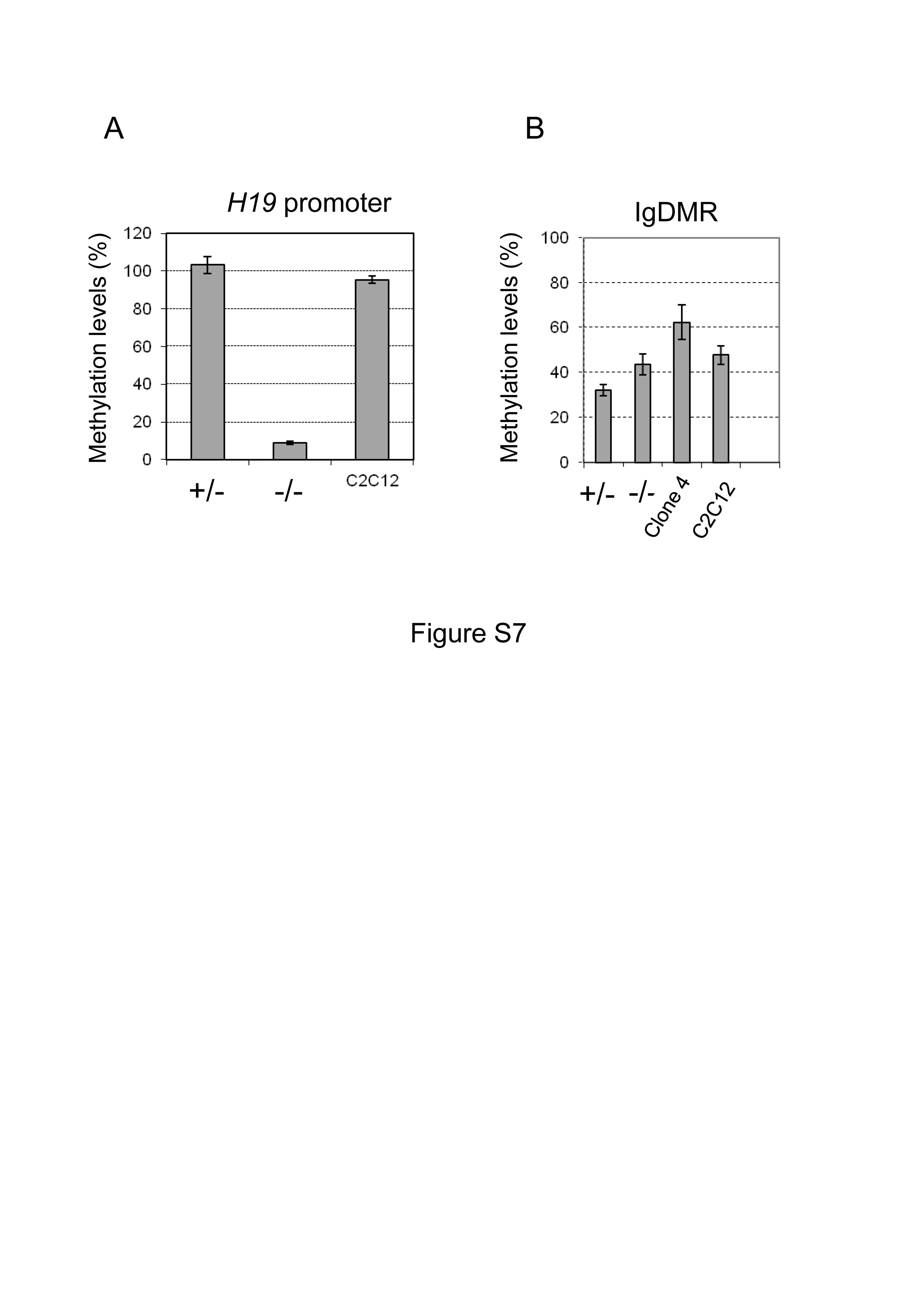

Supplement: Figure S7 — Methylation patterns of H19 promoter and IgDMR (Dlk1/Gtl2 locus on mouse chromosome 12). DNA methylation patterns were analysed in control (+/−) and H19 KO (−/−) myoblasts after 40 passages and in the transfected clone 4 (3 passages after clonal isolation) as well as in C2C12 cells. The methylation pattern of the H19 promoter (A) and the IgDMR (B) were estimated by digestion of the genomic DNA with methylation-sensitive/dependent restriction enzymes (HpaII and McrBC for H19 promoter and IgDMR respectively). Error bars represent s.e.m. of quantifications performed on at least two independent digestions. (TIF) [file pone.0037923.s007.tif]
