# Supplementary material for: H19 Antisense RNA Can Up-Regulate Igf2 Transcription by Activation of a Novel Promoter in Mouse Myoblasts
Source: PLoS One. 2012 May 25;7(5):e37923. doi: 10.1371/journal.pone.0037923 (PMC3360672; doi:10.1371/journal.pone.0037923)
Supplement: Table S1 — Sequences of the qPCR primers used in the present work. Primers indicated in bold were also used in RT reactions. For further details, also see Results and Materials & Methods sections. (DOC) [file pone.0037923.s008.doc]

**Table S1**: qPCR primer sequences.

| Amplicon Names | | Forward Primer Sequences | Reverse Primer Sequences |
| --- | --- | --- | --- |
| mC’ | | 5’-CCGTGTGCTTGAGGCCTCGCCT -3’ | 5’-CAACCTCCCCCCATGAGTCG -3’ |
| mD | | 5’-GGGTGGAAGCGTAGTACCCC-3’ | 5’-CCCTTCCCAGTATCTGTACAGC-3’ |
| mE | | 5’-ATTGTTGGCCCCTTTCCAGGGC-3’ | 5’GTTTGGCATGGGTTCATCTCAGAG3’ |
| mE’ | | 5’-ATTGTTGGCCCCTTTCCAGGGC-3’ | 5’-GCAGGCTCGCGTTGACAAGGAAC-3’ |
| mE’’ | | 5’-GCCCACCAGCAAAGGCTTGCTG-3’ | 5’GTTTGGCATGGGTTCATCTCAGAG3’ |
| mF | | 5’-CCACCTGGCCTTGTCATTCC-3’ | 5’CAGTGGGAGTGGCATAGCTAAC3’ |
| mFb | | 5’-GTTGGGGTTCTGCTTTGCT-3’ | 5’-GCTGTTGGAACAGAAAGGCAG-3’ |
| mF’ | | 5’- CCGGGGAACTGGTGAACTCT -3’ | 5’- GCCTAGTGAGGTCAGGTGGA -3’ |
| mG’ | | 5’-GGTGGGCCCTAGAAAGAGA-3’ | **5’-TGCACACATGCACCCTTCTT-3’** |
| mG’’ | | **5’-AGTCTGTGTGCCCCTTTC-3’** | 5’-GCTGTCACAGATCCCATCAGGC3’ |
| mH | | 5’-TCCCCACTTCCAGAGAGAAA-3’ | 5’-CAGGGGAGGCTAAGAGTCCT-3’ |
| mI1 | | 5’-GGTCTGGCATGACAGACAGAAC-3’ | 5’-AACTTGCGTGGGAGGAGACTG-3’ |
| mI2 | | 5’-GAGCATACTCCCTGCCACAGG-3’ | 5’-CAGACGGCTTCTACGACAAGG-3’ |
| mI3 | | 5’-CCTTGTCGTAGAAGCCGTCTG-3’ | 5’-GGGCAGAAGAGAACTCACCTT-3’ |
| mJ | | 5’-GGCCATGTACTGATTGGTTGAC-3’ | 5’-CCACACCCGGTGCTTCGG-3’ |
| H19 RNA | | 5’-GGAGACTAGGCCAGGTCTC-3’ | 5’-GCCCATGGTGTTCAAGAAGGC-3’ |
| Gapdh | | 5’-ACAGTCCATGCCATCACTGCC-3’ | 5’-GCCTGCTTCACCACCTTCTTG-3’ |
| Igf2 mRNA | | 5’-CACAGAGGGTCCCTCAGCAAG -3’ | 5’-CATAGGAGGCCAGGGAGGTG -3’ |
| Igf2 P0 | | 5’-ATTGACCCAGCCAGCGGATC-3’ | 5’- CTGTACTCTAGTCGCTTCGTAG-3’ |
| Igf2Pm | | 5’-CCCAGTTGAGTGTGTCCTCTG-3’ | 5’-GGTACCCTACTTCTCTCCGAG-3’ |
| Igf2P1 | | 5’-CTCGTCACTTCTCCTACGGTG-3’ | 5’-CCCAGTCGTTTTCCTGGACAC-3’ |
| Igf2P2 | | 5’-GTTCTGTCCCGTCGCACATTC-3’ | 5’-GGTATGCAAACCGAACAGCG-3’ |
| Igf2P3 | | 5’-CTGGACATTAGCTTCTCCTGTG-3’ | 5’-CTGAGGTTGGGTAAGGAGGC-3’ |
| Igf2exon6 | | 5’-ATCGTCCCCTGATCGTGTTA-3’ | 5’-GGAACTGTCCCTGCTCAAGA-3’ |
| PCRa | | **5’-CTGCCTTTCTGTTCCAACAGC-3’** | 5’-CGACTGGAGCACGAGGACACTGA-3’ |
| PCRb | | 5’-GGTCTCTGTTTGTGAGAGCTG-3’ | 5’-CGACTGGAGCACGAGGACACTGA-3’ |
| PCRc | | 5’-CTCACCAGAGTCAGCAGTGTC-3’ | 5’-CGACTGGAGCACGAGGACACTGA-3’ |
| E4_AS3 | | **5’-AGTCTCTCCGGGGCCGTAAGC-3’** |  |
| E4_AS2 | | 5’-GGCCAAAGAGATGAGAAGCAC-3’ | GENE RACER RNA oligo |
| E4_AS1 | | 5’-CAACATCGACTTCCCCACTGG-3’ | GENE RACER RNA oligo |
| BceAI ICR | | 5’-CTCGGACTCCCAAATCAACAAGG-3’ | 5’-ACCCCTGGCCTCATGAAGCCC-3’ |
| McrBC IgDMR | | 5’-ACTCCTGGAGTGAGGGAAGG-3’ | 5’-CAGCTAACCTGAGCTCCATGC-3’ |
| NaeI DMR1 | | 5’-GGGACTCTGTTCCCAGAACC-3’ | 5’-GCTGCAAGCCCTCTGCTAAG-3’ |
| HpaII DMR2 | | 5’-TATGACACCTGGAGACAGTCCG-3’ | 5’-GACGTTTGGCCTCTCTGAACTC-3’ |
| HpaII H19p | | 5’-CCCTTGAGTCCTCCTCCCATC-3’ | 5-GCTTCTGTTGAAAGTTATGCAGCTG-3 |
| 242C19 | | 5’-GTCTCAGGTCTGCTCTCCTGTC-3’ | 5’-TTCTGGTCACCTCTGCTGGATG-3’ |
| ICR H19bis | 5’-GTAAAGAATTTTTTGTGTGTAAAG-3’ | | 5'-CAATACATTCCATAATCACCACAC-3' |
